# Interplay Between Hydrogen Bonding and Electron Transfer in Mixed Valence Assemblies of Triarylamine Trisamides

**DOI:** 10.1002/chem.202203199

**Published:** 2022-12-27

**Authors:** Quentin Sallembien, Paméla Aoun, Sébastien Blanchard, Laurent Bouteiller, Matthieu Raynal

**Affiliations:** ^1^ Institut Parisien de Chimie Moléculaire Equipe Chimie des Polymères Sorbonne Université CNRS 4 Place Jussieu 75005 Paris France; ^2^ Institut Parisien de Chimie Moléculaire Equipe Edifices Polymétalliques Sorbonne Université, CNRS 4 Place Jussieu 75005 Paris France

**Keywords:** charge transport, hydrogen bonds, mixed valence assemblies, supramolecular polymers, triarylamines

## Abstract

Hydrogen‐bonding interactions are assumed to play a critical role in the long‐range transport of light or charge recently observed in supramolecular assemblies of *C*_3_‐symmetrical discotic molecules. Herein, the structure of mixed valence assemblies formed by irradiating triarylamine trisamide (TATA) molecules was determined by multifarious techniques under various conditions with the aim of probing the interplay between the hydrogen bonding network and the rate of electron transport in different states (solution, gel, film). Irradiation was performed under initial states that vary by the degree of association of TATA monomers through hydrogen bonds. Firstly, a significant shift of the N−H and C=O stretching frequencies was observed by FTIR upon irradiation thus revealing an overlooked signature of TATA⋅+ species and interacting mixed valence aggregates. Secondly, gels and films both mostly consist of hydrogen‐bonded TATA polymers but their EPR spectra recorded at 293 K reveal very different behaviors: localized electrons in the gels versus fully delocalized electrons in the films. Hydrogen bonding thus appears as a necessary but not sufficient condition to get fast electron transfer rates and a packing of the TATA monomers particularly suitable for charge transport is assumed to exist in the solid state. Finally, defects in the hydrogen bonding network are detected upon increasing the number of radical species in the mixed valence assemblies present in the film state without impeding the delocalization of the unpaired electrons. A delicate balance between hydrogen bonds and packing is thus necessary to get supramolecular polarons in mixed valence TATA assemblies.

## Graphical abstract

**Not only hydrogen bonds** but also a specific packing arrangement are necessary to get fast electron transfer in mixed valence assemblies of triarylamine trisamides. Defects in the hydrogen bonding network are detected upon increasing the number of radical species in the mixed valence assemblies present in the film state without impeding the delocalization of the unpaired electrons.

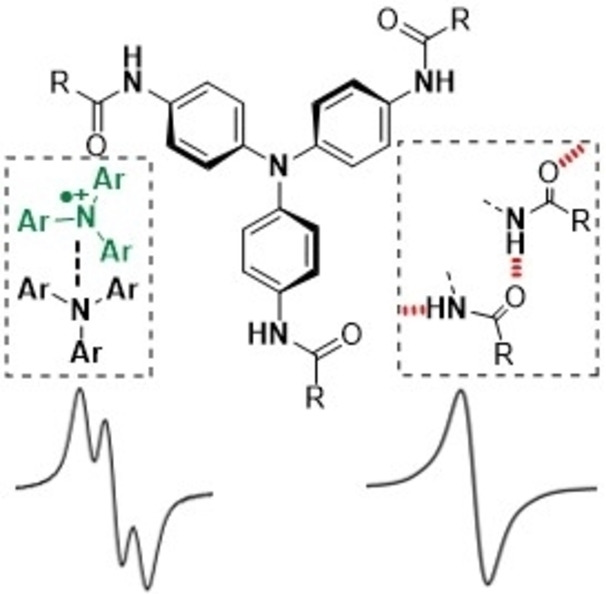

## Introduction

Hydrogen‐bonding interactions have recently emerged as an important structural element for the design of organic semi‐conductor or conductor nanostructures.[^1^, ^2^, ^3^, ^4^, ^5^, ^6^, ^7^, ^8^, ^9^] Notably, *C*_3_‐symmetrical discotic molecules consisting of a central π‐scaffold and hydrogen bonding groups in the side chains generate one‐dimensional helical assemblies^[10]^ that have recently been shown to display impressive long‐range transport of singlet excitons[^11^, ^12^] or electrons.[^13^, ^14^] Long‐range coherent transport is assumed to be the consequence of strong through‐space electronic coupling of the central cores whose stacking is stabilized by the hydrogen‐bonded side chains. The responsiveness of these organic materials to the change in their environment provoked by the delocalization of the exciton or charge might be seen as an advantage that could lead to self‐optimization of their properties through reparation of defects.^[15]^ However, a delicate balance between softness and stability is likely to be needed to generate an adequate packing of the electronically‐coupled monomers.

The redox properties of triphenylamine and its triarylamine (TA) derivatives have been exploited for the design of discrete mixed valence model compounds[^16^, ^17^, ^18^, ^19^, ^20^, ^21^, ^22^, ^23^] and the elaboration of electrochromic materials[^24^, ^25^] amongst other optoelectronic applications. In addition, triarylammonium radicals, such as magic blue, are commonly employed as p‐type redox dopants in semi‐conducting polymers.[^26^, ^27^] Supramolecular polymers assembled from TA‐based monomers thus constitute a particularly interesting class of electroactive molecules with potential implementation in organic electronics and spintronics.^[28]^ Amide functions have been connected through their carbon[^29^, ^30^, ^31^, ^32^] or nitrogen[^13^, ^33^, ^34^, ^35^, ^36^, ^37^, ^38^, ^39^, ^40^, ^41^, ^42^] atom to promote the formation of TA assemblies. Giuseppone and co‐workers disclosed the particularly impressive conductive properties of fibers built on *C*_3_‐symmetrical triarylamine trisamide (TATA) monomers, after their irradiation in chlorinated solvents or in presence of suitable oxidants.^[34]^ Supramolecular delocalized polarons^[43]^ that result from the fast through‐space electron transfer between the connected units make TATA fibers an interesting platform for the design of processable optoelectronic materials.[^40^, ^44^, ^45^, ^46^] As TA compounds do not exhibit such behavior, hydrogen bonding interactions are assumed to be key for the generation of electroactive supramolecular TA‐based polymers. In addition, observation of the fibers by high‐resolution electron microscopy before and after illumination revealed their cooperative self‐healing capacity, that is, the mixed‐valence fibers have higher persistence length than the neutral ones.^[34]^ Whether this optimization process is related to a change in the hydrogen bond network remains to be elucidated.

As it may be anticipated that the packing of the monomers and the nature of the hydrogen bond network differ in solution and in the solid state, we re‐investigated mixed valence assemblies of TATA monomers generated from different initial states. The mixed TATA/TATA⋅+ aggregates have been characterized in solution, gel, and film by means of multifarious techniques with a particular focus on the nature of the hydrogen bond network and the rate of the electron transfer. An unanticipated conclusion of our study is that hydrogen bonding interactions are necessary but not sufficient to get fast electron transfer rates and only the particular packing of TATA molecules in the desolvated state seems to lead to the generation of “polarons” at room temperature. Overall, our results suggest an exquisite balance between packing and hydrogen bonding interactions to achieve full electron delocalization in TA‐based supramolecular polymers.

## Results

### Synthesis of the monomers

**Figure 1:**
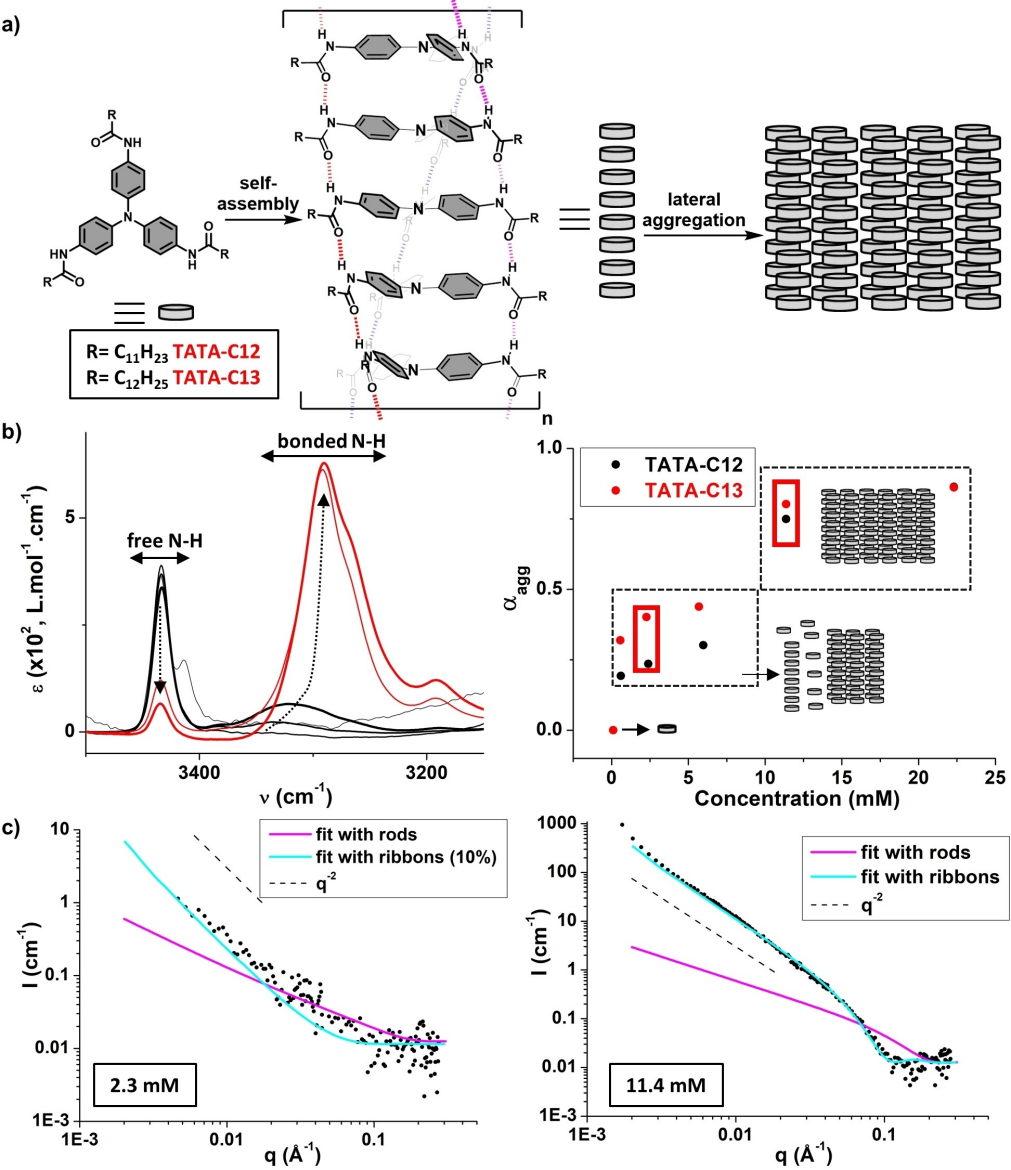
Assemblies in absence of light (solution, 293 K) a) Molecular structures of the monomers investigated throughout this paper and schematic representation of hydrogen‐bonded TATA helices. The orientation of the C=O (in the same direction) and the sense of rotation (left‐handed) have been arbitrarily chosen (an alternative conformation for the C=O has been proposed in the literature).^[29]^ b) Left: Zoom on the N−H region of FTIR analyses of **TATA‐C12** at 0.10, 0.60, 2.39, 5.97, 11.4, and 22.3 mM in CHCl_3_. Dotted arrows indicate the evolution of the FTIR bands as the concentration increases. FTIR spectra of the C=O region and of **TATA‐C13** are shown in Figure S1. The fact that two distinct FTIR bands for free N−H are detected at low concentrations is attributed to the (partial) solvation of TATA monomers by residual water molecules. Right: Degree of association through hydrogen bonds for **TATA‐C12** and **TATA‐C13** as the function of concentration calculated by integration of FTIR band(s) corresponding to free N−H. Conditions selected for probing the effect of illumination are highlighted by red rectangles. c) SANS analyses of **TATA‐C13** at 2.0 g. L^−1^ (2.3 mM, left) and 10.0 g. L^−1^ (11.4 mM, right) in CDCl_3_. The SANS curves have been fitted with a form factor for ribbons (see the Experimental Section for more details).

The previously‐reported TATA monomer, **TATA**‐**C12** (Figure 1),^[34]^ was prepared in one step from commercially available tris(4‐aminophenyl)amine and dodecanoic acid in 76 % yield. The TATA monomer with one additional methylene group in the side chain, **TATA**‐**C13**, was prepared in the same manner (80 % yield) in order to probe a potential odd‐even effect^[47]^ in the assembly behavior. The purity of the two compounds was confirmed by conventional analytical techniques (see the Experimental Section).

### Assemblies in absence of light

Multi‐scale characterization of the self‐assemblies formed by **TATA**‐**C12** and **TATA**‐**C13** have been carried out by means of Fourier‐Transform Infrared (FTIR), UV‐Vis, UV‐Vis‐Near Infrared (NIR) absorption and Nuclear Magnetic Resonance (NMR) spectroscopy as well as Small‐Angle Neutron Scattering (SANS) analyses. Analyses have been conducted in solution and solid states.

FTIR analyses confirm that aggregation in CHCl_3_ is driven by hydrogen bonding interactions between the amide functions from dissociated TATA monomers at 0.1 mM to almost fully hydrogen‐bonded assemblies at 22.3 mM (α_agg_>82 %, Figure 1b). This is accompanied by an increase in the viscosity of the solution above 10 mM, up to formation of opaque self‐supporting gels at 22.3 mM, in agreement with the critical gelation concentration of 17.9 mM previously determined for **TATA**‐**C12**.^[34]^ **TATA**‐**C12** and **TATA**‐**C13** follow similar association trends (Figure 1b) with some differences in the intermediate regime (1–6 mM) which may come from the metastable nature of the aggregates in this regime (see below). Formation of hydrogen‐bonded and excitonically‐coupled monomers in TATA stacks, as schematically represented in Figure 1a, are corroborated by the following diagnostic data: i) FTIR bands at 3290 cm^−1^ and 1655 cm^−1^,[^29^, ^34^, ^36^, ^37^] ii) significant hypochromic and modest hypsochromic (325 nm→322 nm) changes of the lowest energy UV‐Vis absorption band (Figure S2a), and iii) upfield and downfield shifts of aromatic C−H and N−H ^1^H NMR signals, respectively (Figure S2b). SANS analyses in CDCl_3_ show a similar q^−2^ dependence of the scattering intensity at low q values for 2.3 mM and 11.4 mM solutions, albeit with a drastic difference in intensity (Figure 1c). The 11.4 mM SANS curve can be suitably fitted with a form factor for large two‐dimensional objects (such as ribbons) of 54 Å thickness (see the Experimental Section for more details), i.e. approximately two TATA molecules. This indicates that TATA helical stacks tend to aggregate laterally in solution, plausibly upon interdigitation of their alkyl side chains, in agreement with the μm‐scale structures observed by electron microscopy.^[34]^ The low‐intensity SANS signal for the 2.3 mM solution seems to be dominated by a small fraction of ribbons (reasonable fit obtained with 10 %, Figure 1c) but additional smaller aggregates cannot be excluded. These SANS data are consistent with the aforementioned α_agg_ of 38 % and 80 % for 2.3 mM and 11.4 mM solutions, respectively.

**Figure 2:**
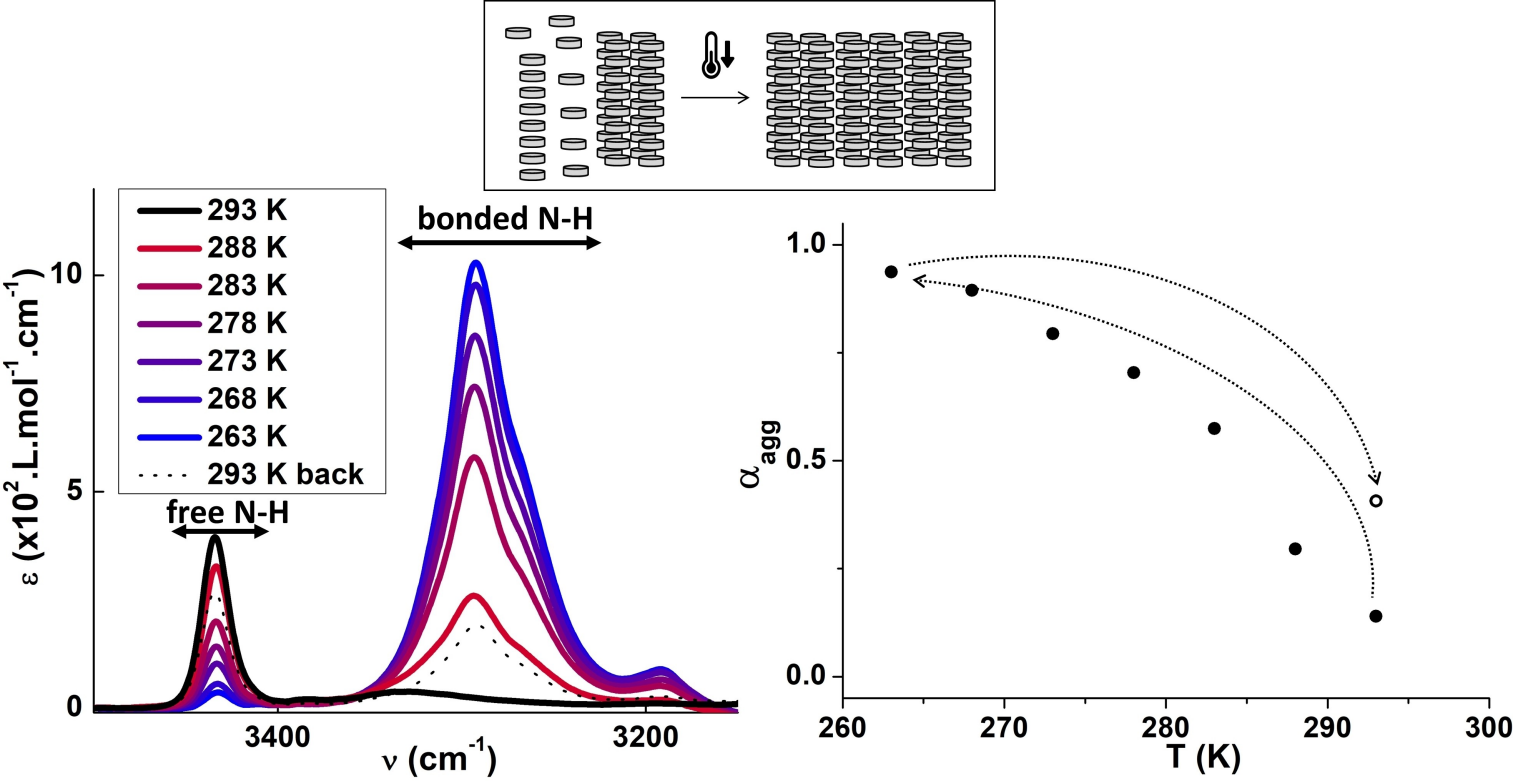
Assemblies in absence of light (solution, 293 K→263 K→293 K). Left: FTIR absorption spectra of a 2.3 mM solution of **TATA‐C13** in CHCl_3_ recorded during cooling process between 293 K and 263 K (red to blue lines, ≈0.4 K.min^−1^, one spectrum recorded every 5 K). Right: Degree of association as function of temperature as deduced from variable‐temperature FTIR spectra. A spectrum was recorded at 293 K after the cooling process (black dotted line and empty circle).

Further examination of the fate of the assemblies present in the intermediate association regime (1–6 mM) reveals their metastable nature. A fluid solution of **TATA**‐**C13** at 2.3 mM that initially contained no more than ≈15 % of hydrogen‐bonded monomers according to FTIR analysis, became more turbid upon standing in the following hours and eventually evolved towards syneresis over days (Figure S3). The corresponding gel was mostly composed of hydrogen‐bonded assemblies as revealed by FTIR analysis. A relatively sharp transition between poorly and ca. fully hydrogen‐bonded TATA monomers is also observed upon lowering the temperature down to 263 K (see FTIR and UV‐Vis‐NIR data in Figures 2 and S3b, respectively). Temperature‐triggered self‐assembly was recently reported for a related TATA monomer within a similar temperature range.^[48]^ The initial state is not recovered upon slow heating since a higher ratio of hydrogen‐bonded monomers appears to be present according to both FTIR and UV‐Vis‐NIR data. Such important thermal hysteresis and the aforementioned observation of gel formation over time are the hallmarks of complex aggregation pathways.[^49^, ^50^] FTIR and UV‐Vis data in the hysteresis regime are virtually identical and thus do not allow to determine the exact nature of the kinetically formed species.^[29]^ Overall, these time‐ and temperature‐dependent experiments suggest a potentially different behavior of this intermediate regime relatively to the approximately fully assembled regime (i.e., ribbons) present at higher concentrations.

**Figure 3:**
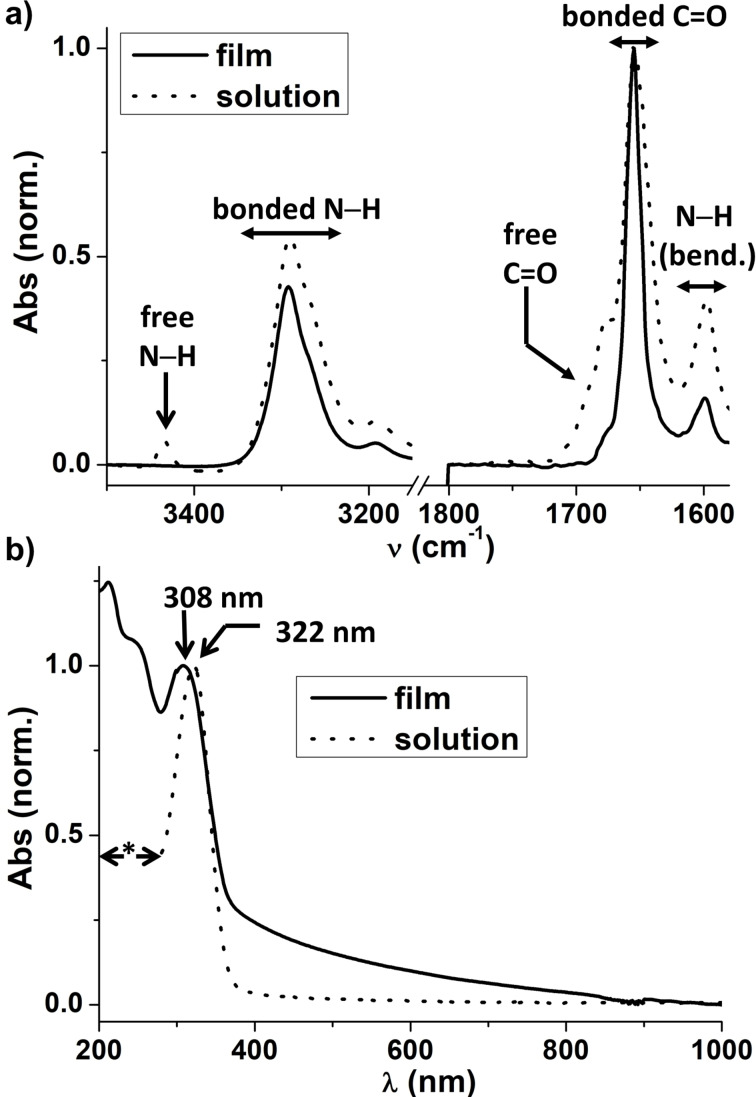
Assemblies in absence of light (comparison of solution and solid states, 293 K). a) FTIR absorption spectra of **TATA‐C12** in the solid (as film) and solution (22.3 mM in CHCl_3_) states. Normalized to the maximum of the band corresponding to bonded C=O (1655 cm^−1^). FTIR spectra of **TATA‐C12** and **TATA‐C13** are compared in Figure S4. b) UV‐Vis‐NIR absorption spectra of **TATA‐C12** in the solid (as film) and solution (11.4 mM in CHCl_3_) states. Normalized to the maximum of the band at ca. 300 nm. The tail observed up to 800 nm in the film state is attributed to scattering. *: signal saturation.

Finally, the assembly behavior was probed in the solid state (under the form of drop casted films) and compared to that observed in solution. FTIR spectra show similar bands in both states but bands related to free amide functions are not detected in the films (Figures 3a and S4). A higher degree of association is thus observed in the film state which is expected given the absence of competing solvent and a plausible further stabilization of the hydrogen‐bonded assemblies by means of dispersion interactions. The lowest energy UV‐Vis absorption band (attributed to π‐π* transition of TATA core) is blue shifted (→308 nm) in solid state (Figure 3b) which can be attributed to a better delocalization of the stacked aromatic rings or a slightly different conformation of the TA unit. The association degree through hydrogen bond can thus be tuned from moderate (intermediate regime in solution), to high (concentrated solutions) and very high (solid state).

### Assemblies in presence of light for 2.3 mM solutions

**Figure 4:**
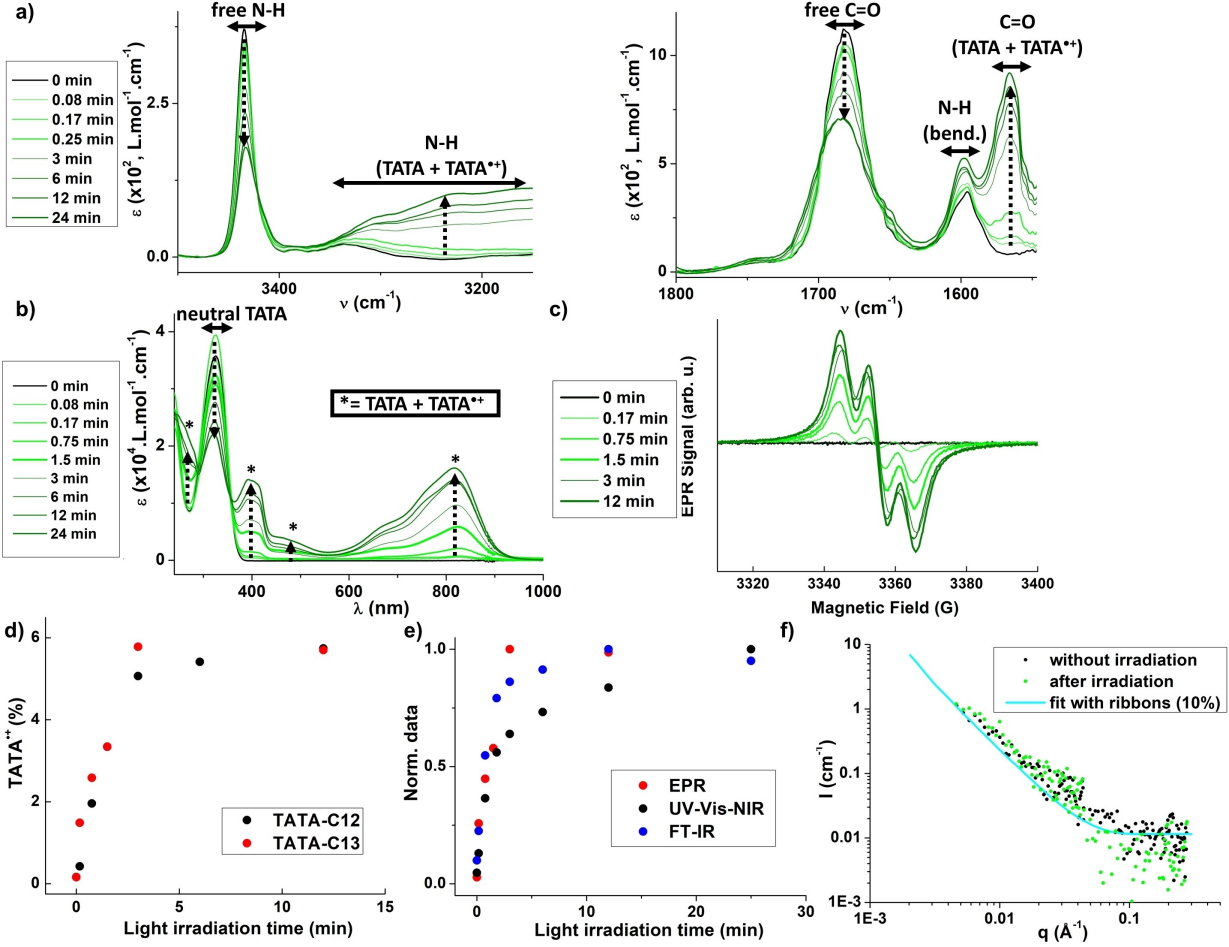
Assemblies in presence of light (intermediate regime, 2.3 mM solutions in CHCl_3_, 293 K). a) FTIR absorption spectra of **TATA‐C12** solution before and after light irradiation (0–24 min), Zoom on the N−H region (left) and C=O regions (right). Arrows serve as a guide to the eyes for the evolution of the main bands upon increasing the irradiation time. b) UV‐Vis‐NIR absorption spectra of **TATA‐C12** solution before and after light irradiation (0–24 min). Arrows serve as a guide to the eyes for the evolution of the main bands upon increasing the irradiation time. c) EPR spectra of **TATA‐C12** solution before and after light irradiation (0–12 min), d) Amount of TATA⋅+ species (as quantified from EPR measurements) as a function of the irradiation time. e) Intensities of EPR data, FTIR data (band at 1566 cm^−1^), and UV–Vis–NIR data (signal at 818 nm) for **TATA‐C12** normalized by their maximal intensity. f) SANS analyses of **TATA‐C13** in CDCl_3_ before irradiation and ca. 7 h after being irradiated for 12 min. At least 1.5 % of radicals are present in the irradiated sample (Figure S7).

Our first goal was to probe the fate of the hydrogen bond network in the presence of light starting from moderately hydrogen‐bonded monomers (20 %< α_agg_<40 %). 2.3 mM solutions of **TATA**‐**C12** or **TATA**‐**C13** in CHCl_3_ were thus irradiated with a LED (λ=385 nm, see the Experimental Section for more details for the illumination setup) for 0−24 min and analyzed at different times by EPR in addition to the aforementioned analytical techniques. Under these conditions, the amount of TATA⋅+ species rapidly reaches a plateau of approximately 6 % after 6 min of irradiation as determined relatively to the integration of the EPR spectrum of a TEMPO standard (Figure 4d). In addition to the detected EPR signal (Figure 4c), the formation of TATA⋅+ species is corroborated by the color change of the solution from colorless to light green and to dark green,[^28^, ^34^, ^37^, ^48^] and the concomitant observation of new UV‐Vis‐NIR signals at 270 nm, 400 nm, 475 nm and 818 nm (Figure 4b). These signals are commonly observed in compounds embedding one^[51]^ or two TA units.^[52]^ Isosbestic points at 289 nm and 357 nm suggest the formation of a single type of either TATA⋅+ species or mixed valence compounds.[^53^, ^54^] The intensity of the signal at 325 nm, representative of neutral species, decreases to ca. 50 % of its initial value after 24 min of light irradiation. As only 6 % of radicals are generated over the same period, it infers that the new UV‐Vis‐NIR signals are related to electronic transitions in mixed valence assemblies.

The generation of radicals is also accompanied by the observation of new bands in FTIR spectra (Figure 4a): a very broad band at approximately 3200 cm^−1^ and a sharp band at 1566 cm^−1^ which increase concomitantly with the decrease of the bands at 3433 cm^−1^ and 1683 cm^−1^, respectively. Again, the former signals decrease by approximately half of their initial intensities. Accordingly, the emerging bands are attributed to the N−H and C=O stretching vibrations of the amide functions of TATA⋅+ species in the mixed valence assemblies of TATA molecules. To the best of our knowledge, these bands have not been reported in the literature and thus constitute new diagnostic signals for TATA⋅+ species.

The decrease of the intensity of the band at 3433 cm^−1^ can thus be ascribed either to the formation of hydrogen bonds and/or to the generation of TATA⋅+ species. The degree of association equal to 52 % after 24 min of irradiation and thus corresponds to a maximal α_agg_ value reached under these conditions. Accordingly, the number of TATA molecules interacting through hydrogen bonds does not drastically increase in the presence of light (α_agg_ maximum goes from 29 % to 52 % in absence and presence of light, respectively) but other non‐covalent interactions between triarylammonium radicals and neutral TATA cannot be excluded.[^33^, ^35^] A significant increase in the fraction of hydrogen‐bonded ribbons would have been detected by scattering techniques which is not the case since SANS analyses show virtually no differences in the scattering profiles between the non‐irradiated and irradiated solutions (Figure 4f).

The evolution of emerging UV‐Vis‐NIR and FTIR bands during illumination is similar to the evolution of the amount of photogenerated TATA⋅+ further corroborating our attribution of these signals (Figure 4e). UV‐Vis‐NIR and FTIR spectra can reasonably be interpreted as the presence of two populations consisting of mixed valence TATA species and neutral TATA molecules. The fact that all aromatic and N−H hydrogens are absent in the ^1^H NMR spectrum of the irradiated sample can be seen either as the formation of long dynamic assemblies[^33^, ^34^, ^35^] (with only a small fraction of hydrogen‐bonded mixed valence molecules in the present case) and/or a slow exchange rate between mixed valence and neutral TATA molecules relatively to their ^1^H NMR frequencies (Figure S5). Virtually identical analyses were obtained with **TATA**‐**C13** indicating that both TATA monomers behave similarly (Figures 4d and S6).

Another striking point of this set of analyses is that EPR spectra, whatever the irradiation time, exhibit a clear three‐line pattern indicative of unpaired electrons localized on one triarylamine unit (Figure 4c). It can be inferred at this point that the high dynamic properties and the low fraction of hydrogen‐bonded TATAs might prevent the delocalization of the unpaired electrons within the mixed valence TATA assemblies. In addition, the persistence time of these radicals is rather limited (Figure S7).

### Assemblies in presence of light for 11.4 mM solutions

**Figure 5:**
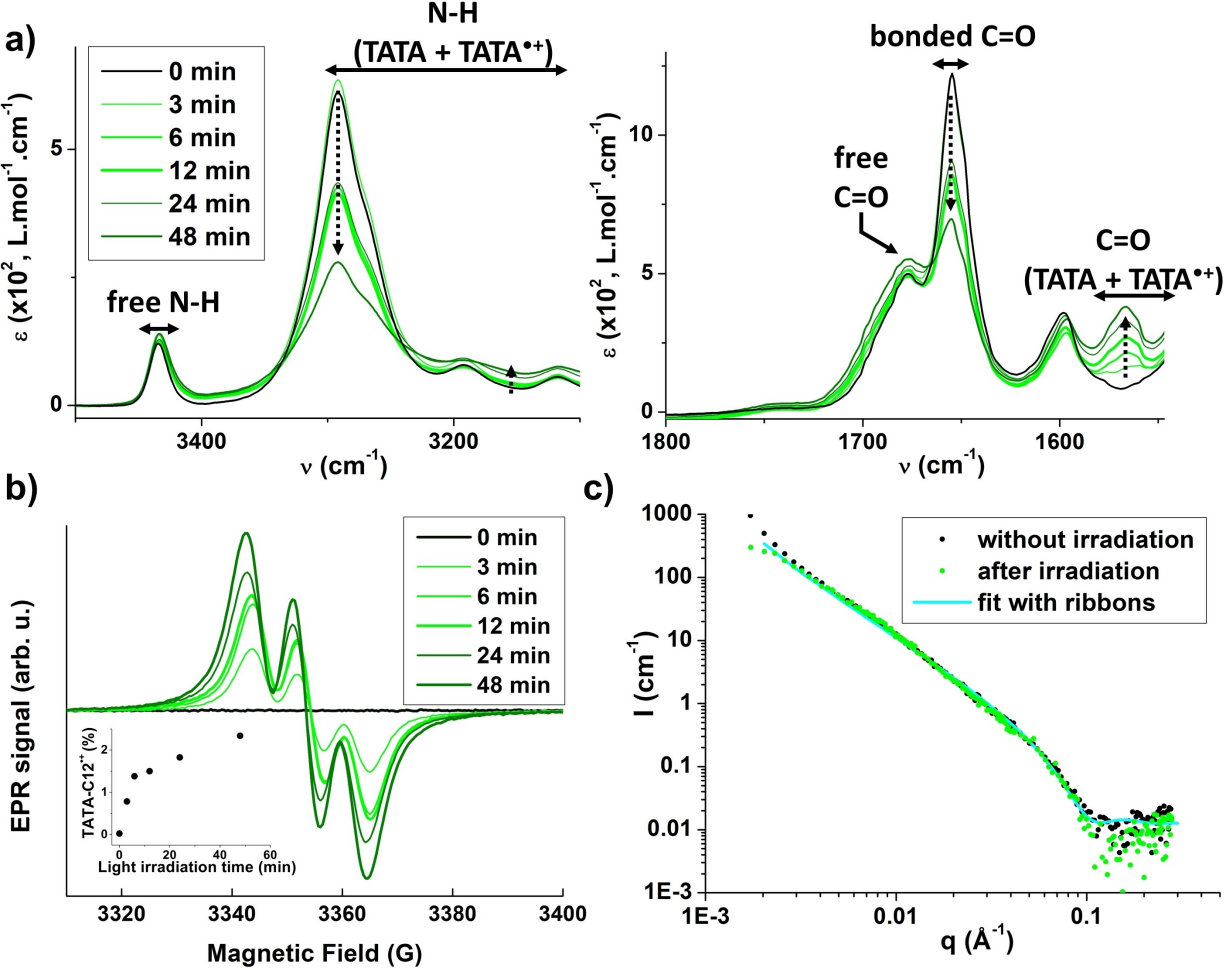
Assemblies in presence of light (concentrated solutions, 11.4 mM solutions in CHCl_3_, 293 K). a) FTIR absorption spectra of **TATA‐C12** before and after light irradiation (0–48 min), Zoom on the N−H region (left) and C=O regions (right). Arrows serve as a guide to the eyes for the evolution of the main bands upon increasing the irradiation time. b) EPR spectra of **TATA‐C12** solution before and after light irradiation (0–48 min), Inset: Amount of **TATA‐C12**⋅+ species as a function of the irradiation time. c) SANS analyses of **TATAC13** in CDCl_3_ before irradiation and ca. 18 h after being irradiated for 12 min. At least 1.3 % of radicals are present in the sample (Figure S8c).

The aforementioned data motivated us to check the influence of radical cations starting with TATA assemblies displaying a higher α_agg_ (75–80 %). The sample which shows a syneresis, that is, it consists of an opaque gel surrounded by a liquid phase (see below), was irradiated for up to 48 min. Despite longer irradiation time, the number of radicals at equilibrium was significantly lower than for the 2.3 mM solution (ca. 2 % maximum, inset of Figure 5b). It may be related to the fact that TATA molecules are less prone to oxidation when located within the ribbons. Mixed valence aggregates exhibit the same characteristic FTIR (Figure 5a) and UV‐Vis‐NIR (Figure S8a) bands but of lower intensities which is consistent with a smaller number of radicals generated under these conditions. The degree of association by hydrogen bonds does not significantly change upon irradiation (ca. 75 %, Figure 5a). Likewise, the only difference in the SANS curve (Figure 5c) is that for the irradiated sample the q^−2^ dependence of the scattering intensity is not maintained until the lowest measured q values, suggesting that the ribbons are shorter after irradiation. However, ribbons remain very large (>35 nm). This is also consistent with the macroscopic observation that the gel is preserved whatever the irradiation times. The fact that a certain degree of re‐organization may occur upon generation of TATA⋅+ species is also reflected by the increase of the intensity of the high energy band in UV‐Vis‐NIR analyses which may indicate a different packing of the TA core in mixed valence aggregates (Figure S8a). Similar to what was observed for 2.3 mM solution, the exchange rate between neutral and TATA⋅+ species is slow relatively to the ^1^H NMR frequencies (Figure S8b).

Rather unexpectedly, EPR spectra (Figure 5b) indicate that the unpaired electron of TATA⋅+ species is localized on a single TA core whatever the extent of illumination, despite the high degree of association of TATA molecules through hydrogen bonds under these conditions. However, radicals are more persistent than for 2.3 mM solutions which may be related to their incorporation (for at least a part of them) inside the ribbons consisting of hydrogen‐bonded TATA molecules (Figure S8c).

### Assemblies in presence of light for wet and dry solids

**Figure 6:**
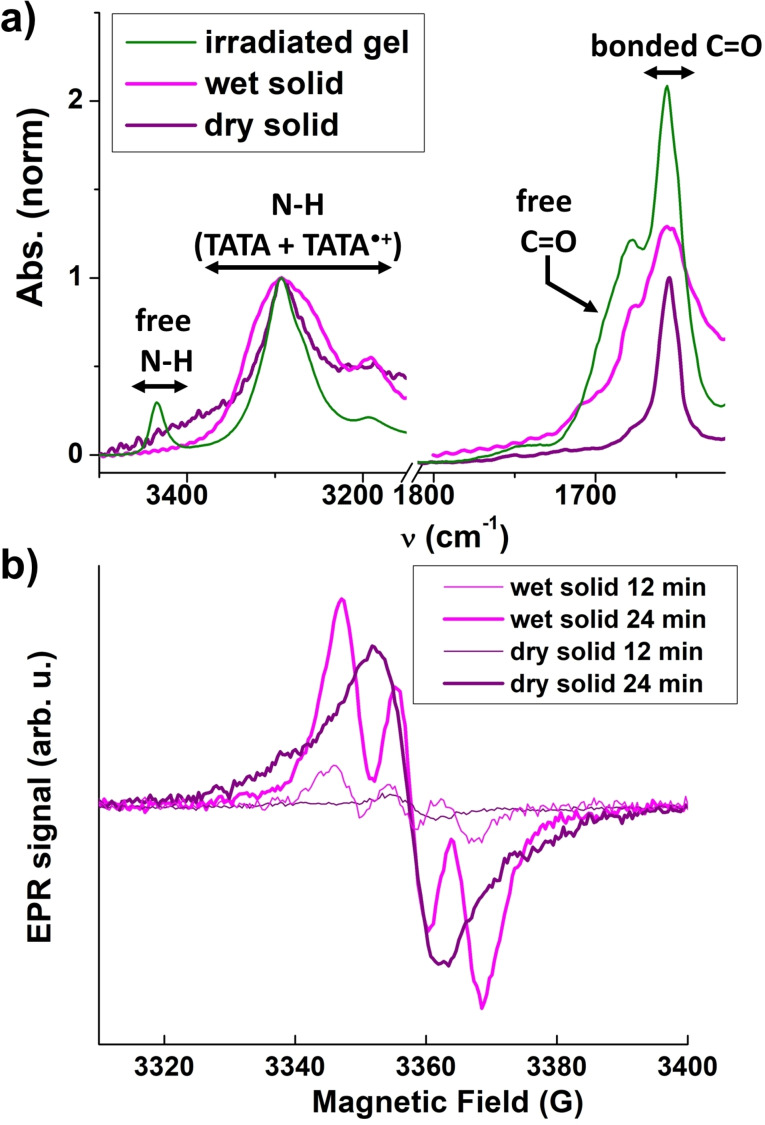
Assemblies in presence of light – wet and dry solid, 293 K. a) FTIR absorption spectra of irradiated gel of **TATA‐C12** (11.4 mM solution in CHCl_3_, 24 min of irradiation) and of the extracted wet and dry solids. Analyses are normalized to the absorbance at 3292 cm^−1^. b) EPR spectra of the wet and dry solids extracted from a 11.4 mM solution of **TATA‐C12** in CHCl_3_ irradiated for 12 min or 24 min.

A possible explanation for the observation of localized electrons in the aforementioned sample could be that the number of radicals actually present in the hydrogen‐bonded assemblies is low and thus their contribution to EPR signals is obscured by isolated TATA⋅+ species. In order to detect exclusively the EPR signal of TATA⋅+ in ribbons, the liquid (hereafter called “the supernatant”) and solid parts of the 24 min irradiated gel were separated by centrifugation and the gel was washed four times with fresh CHCl_3_. The supernatant contains approximately 75 % of the generated TATA⋅+ species which are extracted mostly as poorly hydrogen‐bonded aggregates (α_agg_≈25 %, Figure S9). The CHCl_3_ washings remove mostly neutral TATA species under the form of monomers, thus indicating that the remaining material, hereafter referred to as the wet solid, contains approximately 25 % of the initially light‐generated TATA⋅+ firmly bound within the ribbons (Figures 6a and S9). However, EPR analysis of this wet solid unambiguously shows that unpaired electrons are again localized (Figure 6b). Wet solid with fewer TATA⋅+ species displays the same signature (compare 12 or 24 min, Figure 6b). Combined with the aforementioned data on the irradiated solutions, it thus appears that unpaired electrons in solution and wet solid, whatever the structure adopted by mixed valence TATA assemblies, exhibit low rate of electron transfer according to EPR measurements at 293 K.

A drastically different picture emerges when the solvent of the gel is evaporated. EPR analyses of the thus formed dry solids after 12 and 24 min of irradiation now exhibit a one‐line pattern characteristic of fully delocalized unpaired radicals without energetic barrier to the charge transfer. The origin of the drastic difference in the rate of electron transfer between wet and dry solids is not obvious since both solids have relatively similar FTIR (Figure 6a) and UV‐Vis‐NIR (Figure S9) analyses. Notably, both solids mostly consist of hydrogen‐bonded TATA molecules. However, it is worth noting that the hydrogen bond network appears to be better organized in the fibers present in the dry solid as seen by the narrower N−H and C=O bands of its FTIR spectrum relatively to those exhibited by the irradiated gel and the wet solid (Figure 6a).

### Assemblies in presence of light for films

**Figure 7:**
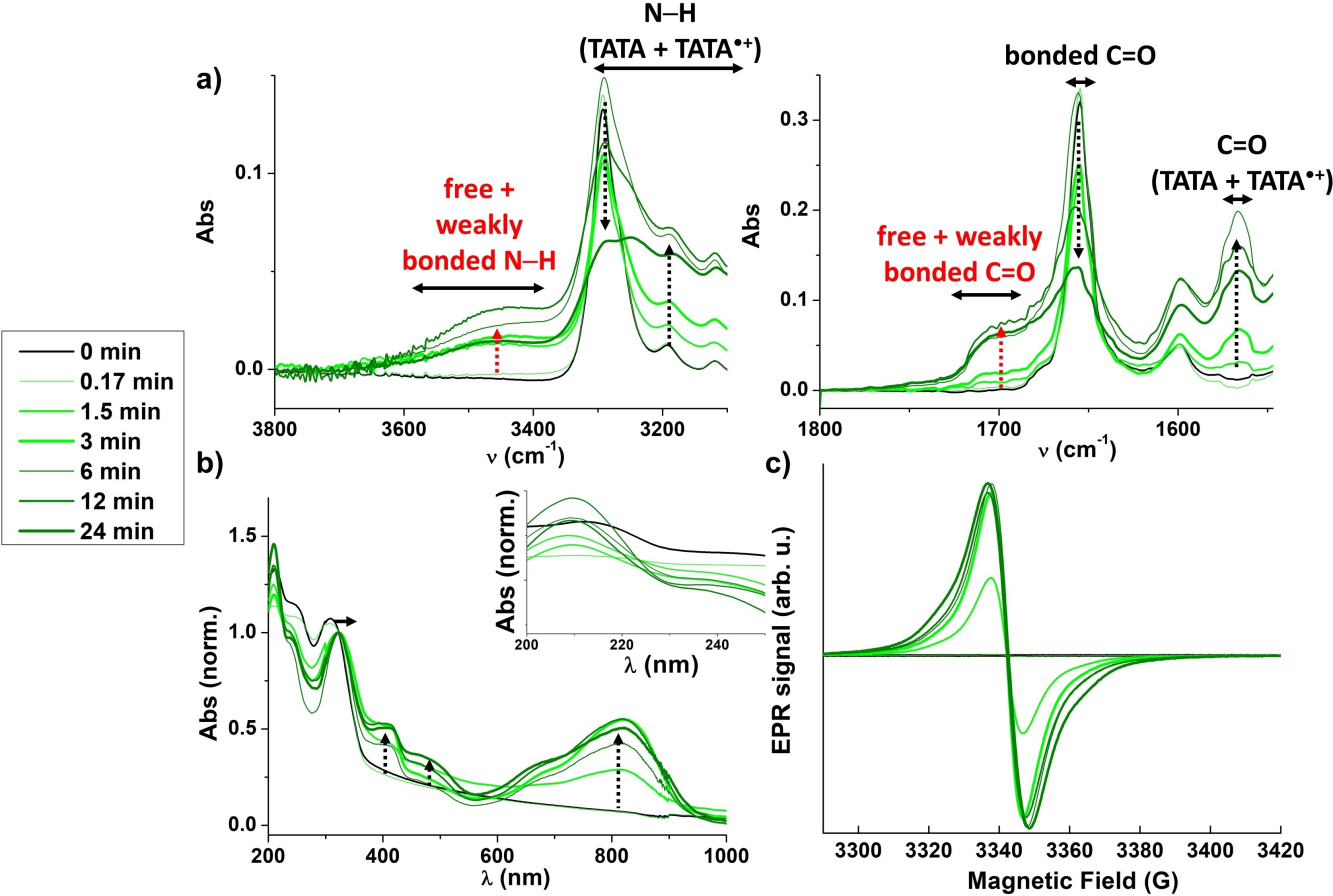
Assemblies in presence of light (films, 293 K). a) FTIR absorption spectra of films obtained by drop coasting of irradiated solutions of **TATA‐C12** at 2.3 mM in CHCl_3_ (0–24 min), Zoom on the N−H region (left) and C=O regions (right). Arrows serve as a guide to the eyes for the evolution of the main bands upon increasing the irradiation time. b) UV‐Vis‐NIR absorption of films obtained by drop coasting of irradiated solutions of **TATA‐C12** at 2.3 mM in CHCl_3_ (0–24 min). Arrows serve as a guide to the eyes for the evolution of the main bands upon increasing the irradiation time. Inset: zoom on the 200–250 nm region. Analyses are normalized to the absorbance at 323 nm. c) EPR spectra of films obtained by drop coasting of irradiated solutions of **TATA‐C12** at 2.3 mM in CHCl_3_ (0–24 min).

The fate of unpaired electrons of TATA•+ species in the film state (obtained by evaporation of a dilute solution) appears particularly interesting to probe for the following reasons: i) it may help to corroborate the difference in the rates of electron transfer between the aforementioned wet and dry solids, ii) interactions between neutral and TATA•+ species are maximized, iii) radicals generated in solution are somewhat enforced to integrate the mixed valence hydrogen‐bonded assemblies upon solvent evaporation thus maximizing the number of radicals in these assemblies compared to solutions or gels and, iv) AFM images previously showed that the persistence length was increased after irradiation.^[34]^ Films were obtained by drop coasting of irradiated solutions of **TATA‐C12** or **TATA‐C13** at 2.3 mM in CHCl_3_, *i.e*. conditions upon which *ca*. 6% of TATA•+ are generated (Figure 4d). The amount of TATA•+ species in the films, which cannot be quantified but is related to the intensity of the EPR signal shown in Figure 7c, reaches a plateau for *ca*. 3‐6 min of irradiation which mirrors well the trend in solution. Unpaired electrons are fully delocalized in the films whatever the irradiation period (EPR spectra, Figures 7c and S10 for **TATA‐C12** or **TATA‐C13**, respectively). EPR spectra of films and dry solids are virtually identical thus validating the aforementioned observation (Figure S11). Analyses of the films after being kept for 20h in the dark show a good persistence of the radicals since only *ca*. 30% of the signals attributed to mixed valence assemblies are lost (Figure S12). This agrees well with the observation by Giuseppone et al. that TATA radicals generated in films are fully delocalized and stable.^[34]^

FT‐IR (Figure 7a) and UV‐Vis‐NIR (Figure 7b) spectra not only show the characteristic signature of mixed valence TATA species but also display additional interesting features. Whilst unirradiated TATA molecules are fully hydrogen bonded, FT‐IR signals attributed to free and/or weakly hydrogen‐bonded N‐H and C=O moieties are detected for irradiated films (Figure 7a, red arrows). More precisely, these bands increase when the amount of triarylammonium radicals in the films increase. In addition, the UV‐Vis band at 308 nm, that was found to be blue shifted in solid state relatively to solution state (*vide supra*, assemblies in absence of light), experiences a red shift upon irradiation (→ 322 nm, horizontal arrow in Figure 7b), *i.e*. it gets displaced to the λ_max_ observed in solution. Likewise, the irradiated films appear to be less diffusive than the non‐irradiated ones or those irradiated for a short period of time (see the tail observed up to 800 nm, Figure 7b). Even though these spectroscopic changes unambiguously reveal that the structure of the films is affected upon incorporation of triarylammonium radicals, it is important to remind that these structural evolutions have no detected influence on the rate of electron transfer, *i.e*. fast delocalization of the electrons is present in the film state whatever the irradiation time (Figure 7c).

## Discussion

**Figure 8:**
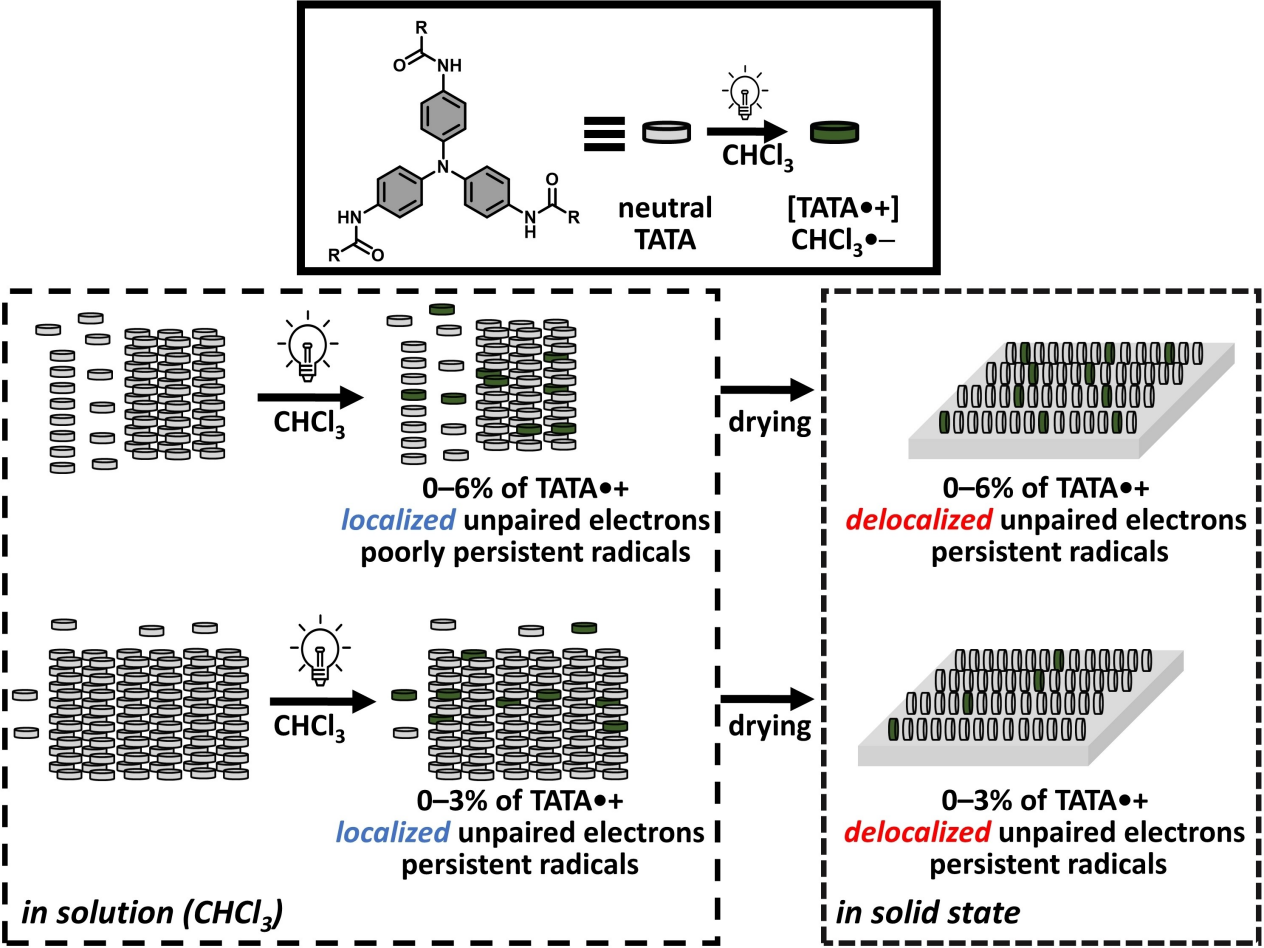
Schematic representation of the mixed valence assemblies and the remarkable features associated with the light‐generated radical cations.

Triarylamine trisamide (TATA) monomers assemble into supramolecular helical polymers thanks to hydrogen‐bonding and aromatic interactions in solution and in solid state.[^29^, ^34^, ^36^, ^37^] Variable‐concentration analyses of **TATA**‐**C12** and **TATA**‐**C13** solutions in CHCl_3_ reveal a relatively broad transition from monomers (≤0.1 mM) to interdigitated hydrogen‐bonded helices (ribbons, ≥11.4 mM) passing through an intermediate metastable state. Prior to this work, it was reasonably assumed that the presence of hydrogen bonds connecting TATA monomers was a sufficient condition to achieve fast electron transfer rate within mixed valence TATA assemblies.^[34]^ However, determination of the structure of mixed valence TATA assemblies by multifarious techniques allows us to describe the more complex interplay between the structure of the assemblies and the extent of charge transfer (relative to EPR time scale) at 293 K, as schematized in Figure 8. Virtually identical analyses have been obtained for **TATA**‐**C12** and **TATA**‐**C13** thus providing further support for the robustness of the reported data^[53]^ and discarding a potential odd/even effect of the side chain.

FTIR and UV‐Vis‐NIR analyses in the intermediate regime reveal distinct signals assigned to neutral and mixed valence species whilst ^1^H NMR analysis is consistent with all molecules being impacted by the electronic interaction between neutral and TATA‐C12⋅+ species. These observations can be made consistent by considering relatively fast and slow exchange rates between neutral and mixed valence species relatively to their FTIR/UV‐Vis‐NIR and ^1^H NMR frequencies, respectively. When considering the relatively small number of radical cations generated under these conditions (up to 6 %) and the limited degree of association though hydrogen bonds (α_agg_ max=52 %), it is probable that triarylammonium radicals and neutral TATA molecules interact through another type of non‐covalent interactions (e.g. dipole‐dipole, aromatic, or van der Waals) in these conditions. However, these interactions are not suitable to allow for a rapid delocalization of unpaired electrons within the detected mixed valence species.

Isolation of the wet solid from the concentrated solution allows us to specifically probe the nature of the unpaired electrons in the solvated ribbons. Surprisingly, electrons appear again to be localized on a single TA core despite the very high degree of association of TATA molecules though hydrogen bonds in the ribbons. It is only after removal of the solvent, in the thus obtained dry solids or films, that unpaired electrons appear to be fully delocalized by means of through space coupling between adjacent triarylamine units in the mixed valence assemblies. Except the drastic difference of their EPR signals, solvated and non‐solvated hydrogen‐bonded ribbons display similar spectroscopic analyses. Note that high energy band(s) in the UV region are not accessible for solvated assemblies which may have precluded identification of band(s) shifts in this region (see inset in Figure 7b). Hence, high degree of association through hydrogen bonds is not a sufficient condition to get supramolecular polarons^[34]^ in the ribbons, with electron transfer rate greater than 10^13^ s^−1^,^[19]^ which likely requires an additional reorganization in the packing of TATA molecules. Subtle effects of the solvent on the structure of supramolecular polymers are commonly observed but are hard to rationalize.^[55]^ In the present case, desolvation might be accompanied by one or several relatively subtle effects such as: i) different packing of aromatic rings of the TA core, ii) change in the conformation of the amide functions (dihedral angle relatively to the aromatic ring, orientation),^[29]^ iii) different distribution of the reduced chloroform molecules (CHCl_3_⋅−)^[35]^ relatively to TATA⋅+. In addition, it cannot be excluded that lateral interactions between supramolecular helices and ribbons, that are favored in the solid state through interdigitation of the alkyl side chains, may also play a role in the extent of the electron transfer rate.

Even though TATA⋅+ species generated upon oxidation of the central TA core and neutral TATA molecules exhibit different geometries, charge transport occurs efficiently in these mixed valence assemblies.[^28^, ^34^] In addition, the increase in the persistence length in the TATA fibers after irradiation indicates that the local reorganization upon integration of triaryl ammonium radicals leads to more rigid fibers.^[34]^ The present study shows that this structural reorganization is probably not accompanied by *stronger* hydrogen bonds, as it could have been reasonably anticipated, but by the emergence of defects in the hydrogen bond network (Figure 7a). Our results suggest that the healing process in mixed valence TATA fibers is supported by *a weakening* of the hydrogen bond network which is probably over‐compensated by an optimal stacking of the TATA core. Of particular interest is the fact that neither disassembly of the fibers[^34^, ^40^] nor lower electron transfer rate have been observed in the present work probably because of the relatively small amount of generated TATA⋅+ species at equilibrium (6 % maximum).

## Conclusion

The remarkable charge‐transport properties of triarylamine trisamide (TATA) fibers are undoubtedly related to the ability of these monomers to assemble through hydrogen bonds yielding optically‐triggered supramolecular polarons.[^28^, ^34^, ^44^, ^45^, ^46^] However, precise investigation of the structure of the mixed valence assemblies formed upon irradiation of two model TATA molecules reveal a more intricate interplay between the hydrogen bond network and the rate of the electron transfer in the fibers. FTIR analyses appear particularly insightful since it not only offers a precise picture of the hydrogen bond network but also provides an overlooked signature of TATA⋅+ species and the related mixed valence assemblies. Amongst the different investigated conditions, a fast regime for the electron transfer rate (relatively to EPR at 293 K) was observed only for desolvated assemblies in the solid state. Although the degree of association through hydrogen bond for ribbons in solution is very high, the electron transfer rate is significantly lower since only localized electrons are detected by EPR. Thus, not only hydrogen bonds but also an efficient packing of the TA units is required to get supramolecular polarons. Finally, the structural re‐organization related to the healing process triggered by the integration of TATA⋅+ species within TATA fibers and delocalization of their charge is more complex than anticipated. Weakening of hydrogen bonds is likely necessary to maintain efficient packing between neutral and TATA⋅+ species. A proper rationalization of the role of hydrogen bonds may lead to the development of more efficient and more structurally complex organic semi‐conductor and conductor nanostructures.

## Experimental Section

**Materials**: n‐Dodecanoic acid, n‐tridecanoic acid, *N*‐(3‐dimethylaminopropyl)‐*N*‐ethylcarbodiimide hydrochloride (EDC⋅HCl) and 4‐(dimethylamino)pyridine (DMAP) were purchased from Acros, Alfa Aesar, Sigma‐Aldrich, or Fluorochem suppliers and used as received. Tris(4‐aminophenyl)amine (>98 %) was ordered from TCI and used as received. Unless otherwise noted, chromatography‐grade solvents were used as received. Anhydrous *N*,*N*‐dimethylformamide (99.8 %, dry over molecular sieves) was purchased from Acros Organics. Chloroform was filtered over basic alumina, distilled over CaH_2_ and stored under argon atmosphere in a Schlenk flask before use in all of the experiments. THF‐d_8_ was bought from Eurisotop and used without further purification. CDCl_3_ was bought from Eurisotop, distilled over CaH_2_ and stored under argon atmosphere in a Schlenk flask.

**Methods**: *Nuclear Magnetic Resonance (NMR) analyses*: NMR spectra of TATA monomers were recorded in THF‐d_8_ on a Bruker Avance 300 or 400 spectrometer and calibrated to the residual solvent peak: ^1^H: 3.58 and 1.72 ppm; ^13^C: 67.21 and 25.31 ppm. Peaks are reported with their corresponding multiplicity (s: singlet; d: doublet; t: triplet; m: multiplet), integration and *J* coupling constant given in Hertz. ^1^H NMR spectra of the TATA assemblies were recorded in CDCl_3_ prior to and after illumination on a Bruker Avance 400 spectrometer and calibrated to the residual solvent peak (7.26 ppm).

*High Resolution Mass Spectrometry (HRMS) analyses*: Exact mass measurements were obtained on TQ R30‐10 HRMS spectrometer by ESI+ ionization and are reported in *m/z* for the major signal.

*Fourier‐Transform Infrared (FTIR) analyses*: FTIR measurements were performed on a Nicolet iS10 spectrometer. Solution spectra were measured in CaF_2_ cells of various pathlengths (0.5, 1 and 2 mm, according to the concentration) and were corrected for air, solvent and cell absorption. Spectra for solutions were reported in extinction molar coefficient (L.mol^−1^.cm^−1^) as a function of the wavenumber. Films and gels were measured on KBr cells and corrected for air and cell absorption. Attenuated total reflectance (ATR) FTIR spectra for solids were collected on a Bruker tensor 27 FTIR spectrometer equipped with the ATR platinum module, with a detector RT‐DLaTGS. The OPUSv5.5 software (Bruker Optics, Germany) was set up with the following parameters: the spectral resolution was fixed to 4 cm^−1^, the number of scans to 32 and the selected spectral range between 4000 and 400 cm^−1^. The main peaks were reported as m: medium or s: strong.

*UV–Vis analyses*: UV‐Vis absorption spectra were performed on a Jasco J‐1500 spectrometer equipped with a Peltier thermostated cell holder and a Xe laser. Data were recorded at 293 K in CHCl_3_ between 800 and 270 nm and extracted after correction of the absorption of air, solvent, and cell contribution at the same temperature. The pathlength of the cell was adapted as follows: 1 mm rectangular quartz cell for 0.1 mM solution, 0.1 mm Starna© cylindrical closed quartz cell for 2.3 mM solution and 0.05 mm Starna© cylindrical closed quartz cell for 11.4 mM solution. Spectra were reported in extinction molar coefficient (L.mol^−1^.cm^−1^) as a function of the wavelength.

*UV–Vis–NIR analyses*: UV–Vis–NIR absorption spectra were measured on a Varian Cary 5000 spectrophotometer. Data were recorded with the following parameters: double beam mode, 600 nm.min^−1^ sweep rate, 1 nm data pitch, 2 nm bandwidth, and between 1500 and 240 nm (for solutions) or between 1500 and 200 nm (for films) and automatically extracted after correction of the absorption of air, solvent, and cell contribution at the same temperature. Spectra are shown only between 1000 and 240 or 200 nm since no signals have been detected in the 1000–1500 nm region.^[53]^ Spectra were corrected for the sudden change in intensity that occurs at 900 nm due to change of the light source. Spectra were reported in extinction molar coefficient (L.mol^−1^.cm^−1^) as a function of the wavelength. Films and gels were measured on lamellar quartz cells and corrected for air and cell absorption.

*Small‐angle neutron scattering (SANS) analyses*: SANS measurements were made at the LLB (Saclay, France) on the PA20 instrument, at three distance‐wavelength combinations to cover the 1.72 ⋅ 10^−3^ to 0.30 Å q^−1^ range, where the scattering vector q is defined as usual, assuming elastic scattering, as q=(4π/λ)sin(θ/2), where θ is the angle between incident and scattered beam. Data were corrected for the empty cell signal and the solute and solvent incoherent background. A light water standard was used to normalize the scattered intensities to cm^−1^ units. The data was fitted with the DANSE software SasView with a form factor for flat objects of finite thickness and infinite lateral dimensions. The thickness obtained from the fit (54 Å) corresponds to roughly twice the diameter for extended **TATA**‐**C13** molecule (30 Å).^[37]^

*Electron Paramagnetic Resonance (EPR) analyses*: X‐band EPR spectra were recorded in non‐saturating conditions on a Bruker ELEXSYS 500 spectrometer at 293 K. Typical experimental conditions were: 9.396 GHz microwave frequency, 2 mW microwave power, 0.1 mT modulation amplitude, 100 kHz modulation frequency. Samples for EPR measurements were prepared by introducing solutions or gels into the EPR tubes with a metal needle or directing forming the films into the EPR tubes (drying with nitrogen flow). Simulations were performed using the EasySpin program.^[56]^ 2,2,6,6‐tetramethyl‐piperidin‐1‐oxyl (TEMPO) was used as a reference to determine the quantity of spins per molecule. Classical representation of the experimental EPR spectra as the derivative of the absorption signal as a function of the magnetic field is used: thus, small variations of the microwave frequencies applied for the detection lead to small shifts between the spectra, the g‐values being however identical. Simulation of the three‐line pattern EPR signals (localized unpaired electrons): g=2.0015; A=8.6G (24 MHz). For one‐line pattern EPR signals (delocalized unpaired electrons): g=2.0014.

*Illumination conditions*: The illumination setup is composed of a LED (optical power: 1650 mW centered on 385 nm), a condenser lens, a linear polarizer and a quarter‐wave Fresnel rhomb retarder or a quarter‐wave plate, all aligned on an optical bench. A linear polarizer and a photodiode, linked to an optical power meter console, served to measure the surface optical power of the light emitted by this setup which was equal to ca. 8 mW.cm^−2^. The setup and sample were placed in an opaque box in order to avoid illumination by external sources. The solutions were inserted in a 2 mm rectangular quartz cell and the cell was placed at 1–3 cm from the light source. After the desired irradiation time, the solution was moved to the required container (quartz cell, CaF_2_ cell, EPR tube) for characterization.

*Preparation of the solutions*, *gels and films*: The desired amount of **TATA**‐**C12** or **TATA**‐**C13** was weighed as well as CHCl_3_ or CDCl_3_ and the obtained suspension was gently heated in order to get a homogeneous solution. The solution was cooled down to room temperature and analyzed directly (for analyses without illumination) or transferred to a 2 mm rectangular quartz cell (for analyses requiring sample illumination). Thin films were obtained by evaporation of a CHCl_3_ solution of **TATA**‐**C12** or **TATA**‐**C13** onto KBr cells or lamellar quartz cells.

*Synthesis of TATA monomers*: **TATA**‐**C12** was previously reported through a different route^[34]^ and analyzed in MeOD/C_7_D_8_ (5 : 3 mixture) which hampered the observation of the amide N−H. Accordingly, we reported a new synthetic protocol for **TATA**‐**C12** and its characterization in THF‐d_8_.

*Synthesis of **TATA***‐***C12***: n‐Dodecanoic acid (1.56 g, 7.76 mmol, 4.5 equiv), EDC ⋅ HCl (1.49 g, 7.76 mmol, 4.5 equiv) and DMAP (0.99 g, 8.11 mmol, 4.7 equiv) were dissolved in 75 mL of anhydrous *N*,*N*‐dimethylformamide at room temperature under argon for 10 minutes. Tris(4‐aminophenyl)amine (500 mg, 1.72 mmol, 1.0 equiv) was then added to the solution and the mixture was stirred for 2 days. The reaction mixture was poured into brine (100 mL) and extracted with non‐stabilized THF (3 ⋅ 150 mL). The organic layer was washed with brine (4 ⋅ 150 mL), saturated NaHCO_3_ (up to pH=8), dried over MgSO_4_ and evaporated under vacuum, to yield the crude product, which was further recrystallized in ethanol, to yield **TATA**‐**C12** as an off‐white solid (1.10 g, 76 % yield). ^1^H NMR (THF‐d_8_, 300 MHz, 300 K): δ (ppm)=8.80 (s, 3H), 7.48 (AB system, d, *J*=8.4 Hz, 6H), 6.90 (AB system, d, *J*=8.5 Hz, 6H), 2.25 (t, *J*=7.4 Hz, 6H), 1.66 (quintet, *J*=7.0 Hz, 6H), 1.38–1.11 (m, 48H), 0.89 (t, *J*=6.5 Hz, 9H). 13 C{^1^H} NMR (THF‐d_8_, 100.6 MHz, 300 K): δ (ppm)=171.17, 144.39, 135.95, 124.82, 120.91, 37.85, 33.03, 30.78, 30.75, 30.70, 30.64, 30.48 (⋅ 2), 30.45, 26.66, 23.71, 14.59. FT–IR (ATR, cm^−1^): 817 (m), 1272 (m), 1316 (m), 1406 (m), 1467⋅, 1508 (s), 1600 (m), 1655 (s), 2851 (m), 2921 (m), 3293 (m). HRMS (ESI, *m/z*): calculated for C_54_H_84_N_4_O_3_H, [M+H]^+^: 837.6616, found: 837.6619.

*Synthesis of **TATA***‐***C13***: n‐Tridecanoic acid (1.67 g, 7.80 mmol, 4.5 equiv), EDC ⋅ HCl (1.496 g, 7.80 mmol, 4.5 equiv) and DMAP (1.0 g, 8.15 mmol, 4.7 equiv) were dissolved in 75 mL of anhydrous *N*,*N*‐dimethylformamide at room temperature under argon for 10 minutes. Tris(4‐aminophenyl)amine (504 mg, 1.73 mmol, 1.0 equiv) was then added to the solution and the mixture was stirred for 2 days. The reaction mixture was poured into brine (100 mL) and extracted with non‐stabilized THF (3 ⋅ 150 mL). The organic layer was washed with brine (4 ⋅ 150 mL), saturated NaHCO_3_ (up to pH=8), dried over MgSO_4_ and evaporated under vacuum, to give the crude product, which was further recrystallized in ethanol and ethyl acetate, to yield **TATA**‐**C13** as an off‐white solid (1.22 g, 80 % yield). 1H NMR (THF‐d_8_, 300 MHz, 300 K): δ (ppm)=8.84 (s, 3H), 7.47 (AB system, d, *J*=9.0 Hz, 6H), 6.90 (AB system, d, *J*=8.9 Hz, 6H), 2.25 (t, *J*=7.4 Hz, 6H), 1.66 (quintet, *J*=7.2 Hz, 6H), 1.43–1.09 (m, 54H), 0.88 (t, *J*=7.0 Hz, 9H). ^13^C{^1^H} NMR (THF‐d_8_, 100.6 MHz, 300 K): δ (ppm)=171.07, 144.39, 135.94, 124.83, 120.88, 37.86, 33.04, 30.80 (⋅ 2), 30.76, 30.71, 30.65, 30.48 (⋅ 2), 26.65, 23.73, 14.59. FT–IR (ATR, cm^−1^): 519 (m), 721 (m), 818 (m), 1244 (m), 1269 (m), 1313 (m), 1404 (m), 1466 (m), 1504 (s), 1599 (m), 1655 (m), 2851 (m), 2920 (m), 3292 (m). HRMS (ESI, *m/z*): calculated for C_57_H_90_N_4_O_3_Na, [M+Na]^+^: 901.6905, found: 901.6909.

## Conflict of interest

The authors declare no conflict of interest.

1

## Supporting information

As a service to our authors and readers, this journal provides supporting information supplied by the authors. Such materials are peer reviewed and may be re‐organized for online delivery, but are not copy‐edited or typeset. Technical support issues arising from supporting information (other than missing files) should be addressed to the authors.

Supporting Information

## Data Availability

The data that support the findings of this study are available from the corresponding author upon reasonable request.
