# Supplementary material for: Interplay Between Hydrogen Bonding and Electron Transfer in Mixed Valence Assemblies of Triarylamine Trisamides
Source: Chemistry. 2022 Dec 27;29(9):e202203199. doi: 10.1002/chem.202203199 (PMC10107863; doi:10.1002/chem.202203199)

Chemistry–A European Journal

Supporting Information

Interplay Between Hydrogen Bonding and Electron Transfer in Mixed Valence Assemblies of Triarylamine Trisamides

Quentin Sallembien, Paméla Aoun, Sébastien Blanchard, Laurent Bouteiller, and Matthieu Raynal*

Contents

Supplementary Charts, Figures and Tables (Figures S1-S12) ______________________________ *2*

NMR spectra ____ *11*

Supplementary Charts, Figures and Tables (Figures S1-S12)

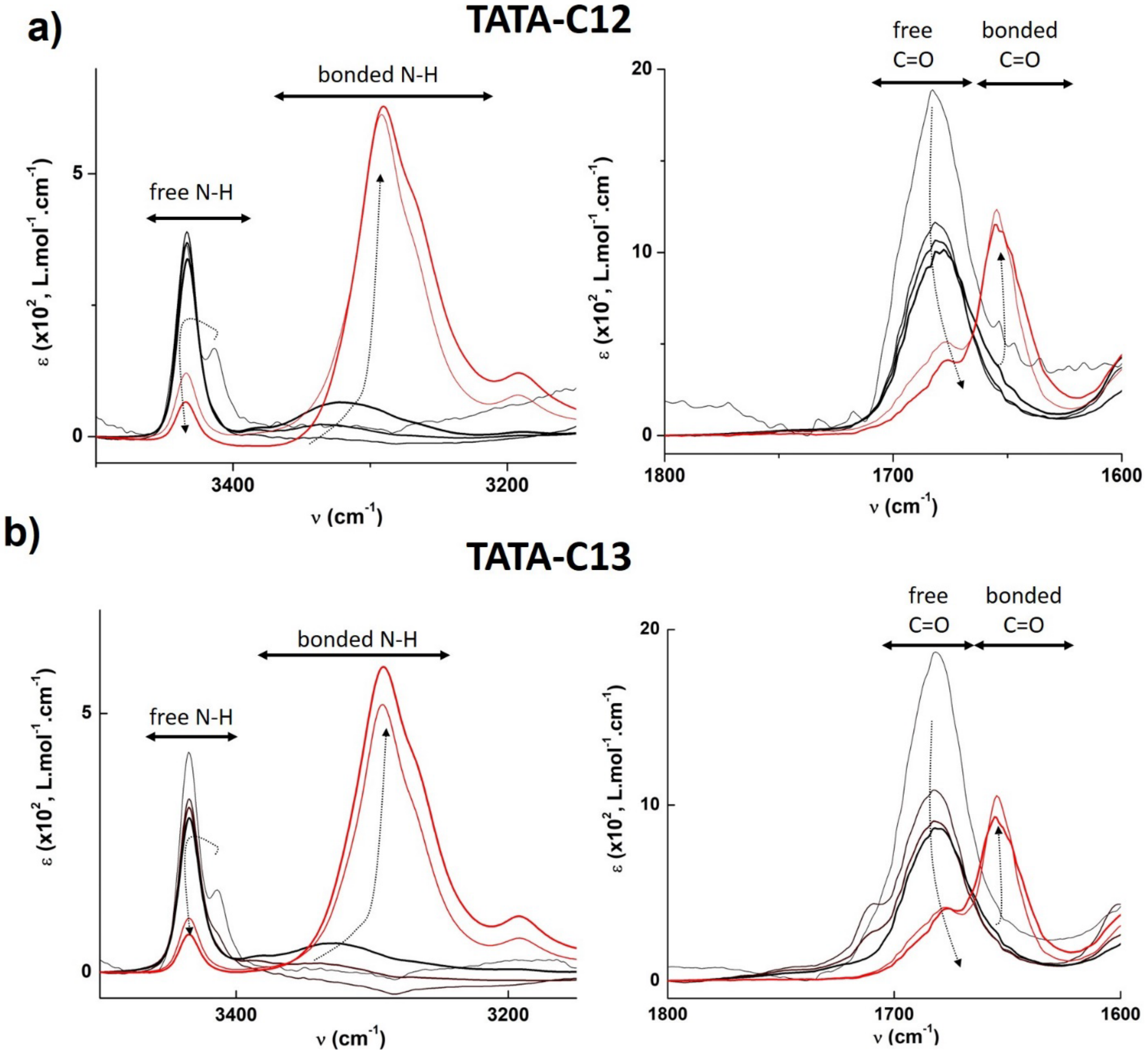

Figure S1 a) Zoom on the N—H and C=O regions of FT-IR analyses of **TATA-C12** at 0.10, 0.60, 2.39, 5.97, 11.4, and 22.3 mM in $CHCl_3$. Dotted arrows indicate the evolution of the FT-IR bands as the concentration increases. N—H region is shown in the main text as Figure 1b, left. b) Zoom on the N—H and C=O regions of FT-IR analyses of **TATA-C13** at 0.10, 0.57, 2.27, 5.69, 11.4, and 22.3 mM in $CHCl_3$. Dotted arrows indicate the evolution of the FT-IR bands as the concentration increases.

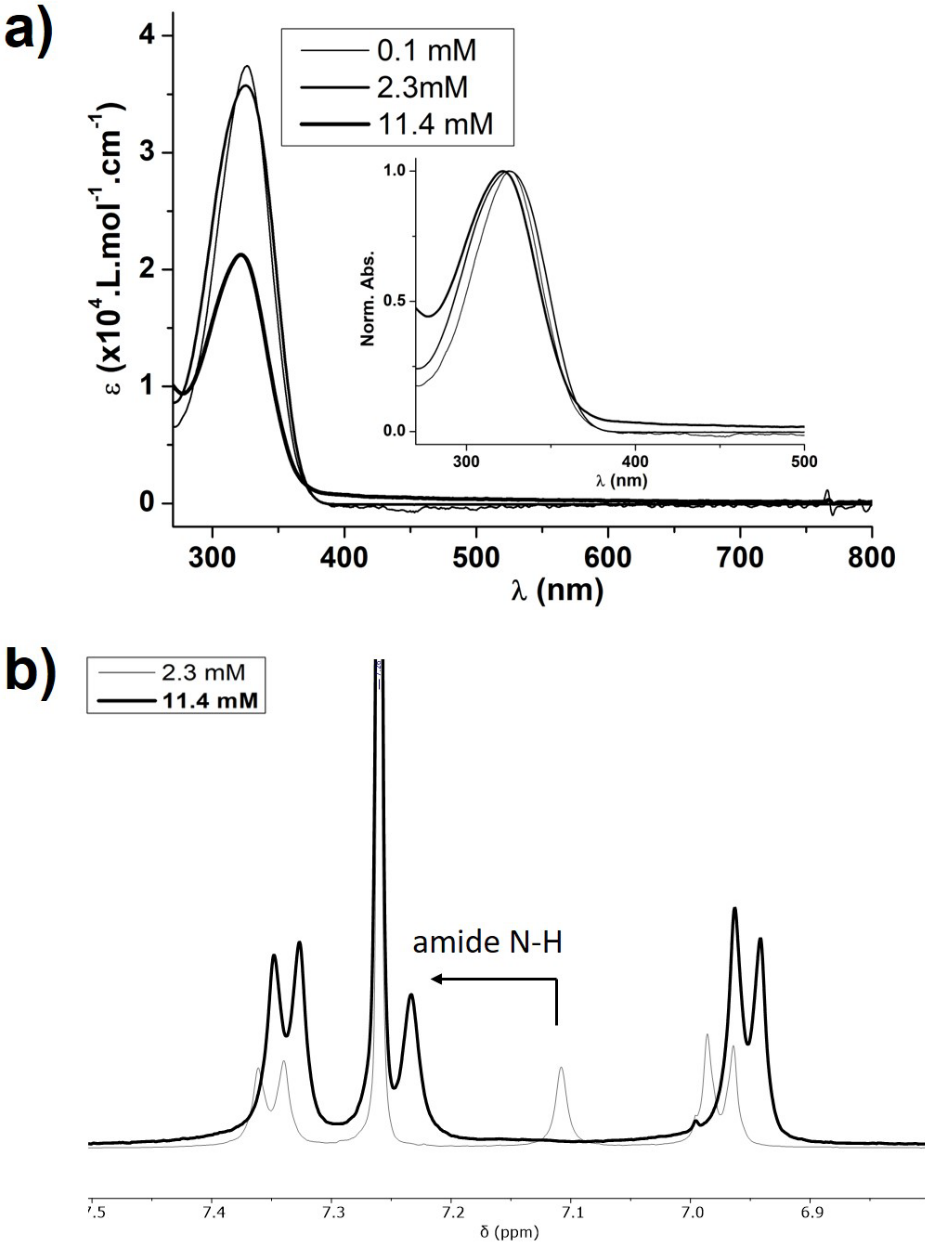

Figure S2 a) UV-Vis absorption spectra of **TATA-C12** in $CHCl_3$ at various concentrations. Inset: UV-Vis spectra normalized to the intensity of the λ_{max}. Hypochromic (decrease of intensity from 3.8×10^{-4} to 2.1×10^{-4} L.mol^{-1}.cm^{-1}) and hypsochromic (shift of the λ_{max} from 325 to 322 nm) absorption changes are observed upon increasing the concentration. Scattering by TATA ribbons is also detected (baseline shift) for the 11.4 mM solution. b) ^{1}H NMR spectra of **TATA-C12** in $CDCl_3$ at 2.3 mM and 11.4 mM. The NMR spectra are normalized to the maximum intensity of the residual $CHCl_3$ signal.

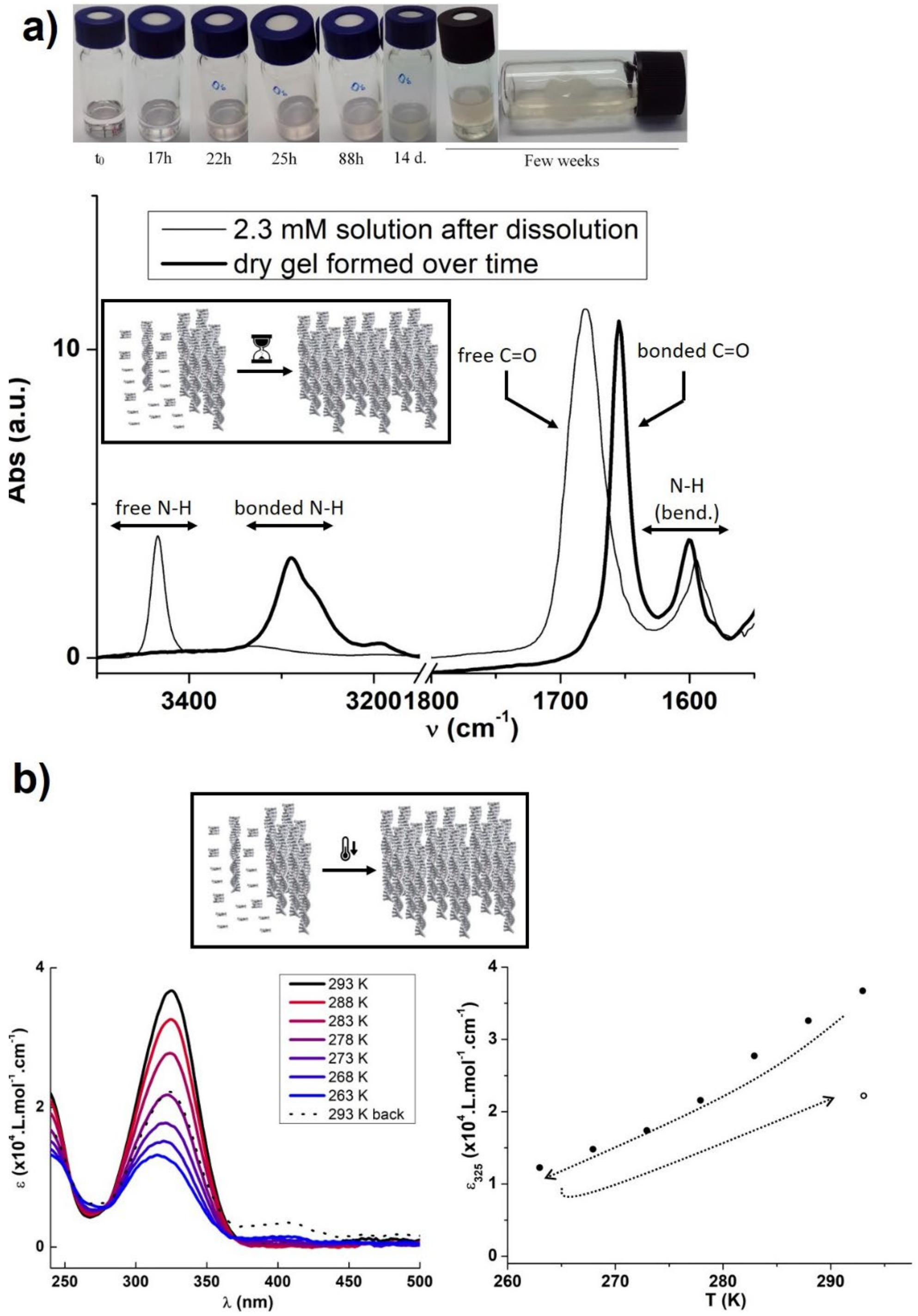

Figure S3 a) Assemblies formed upon standing of a 2.3 mM solution of **TATA-C13** in $CHCl_3$. Pictures of the sample at different times. Comparison of the FT-IR spectra of the initial solution and the gel formed over time. b) UV–Vis absorption spectra recorded during a cooling-heating cycle between 293 K and 263 K, with a spectrum recorded every 5 K (0.5 $K.min^{-1}$). Extinction molar coefficient at 325 nm as a function of temperature as deduced from variable-temperature UV-Vis spectra. A spectrum was recorded at 293 K after the cooling process (black dotted line and empty circle).

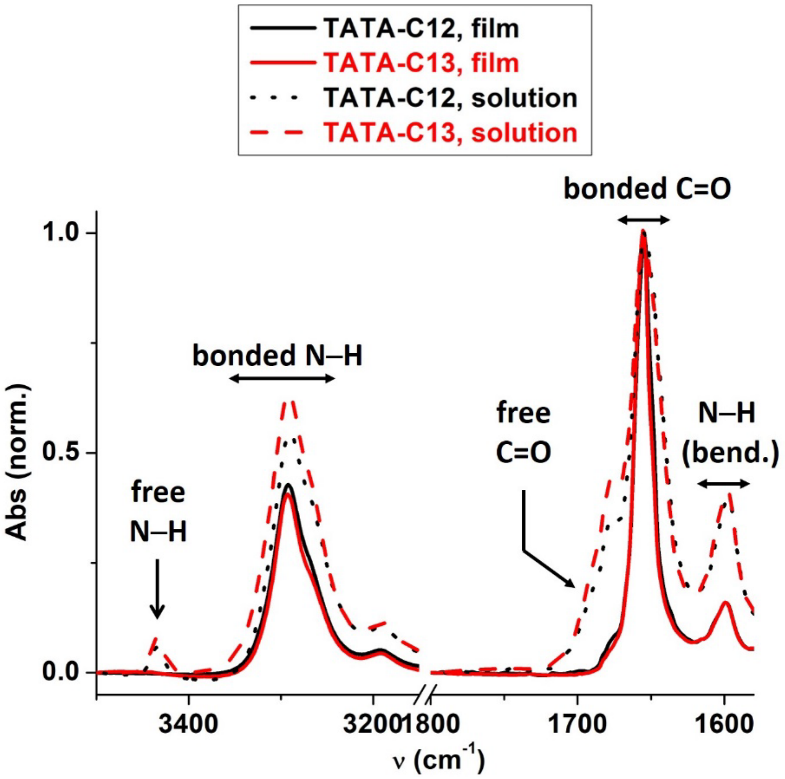

Figure S4 FT-IR absorption spectra of **TATA-C12** and **TATA-C13** in the solid (as film) and solution (22.3 mM in $CHCl_3$) states. Normalized to the maximum of the bonded C=O band.

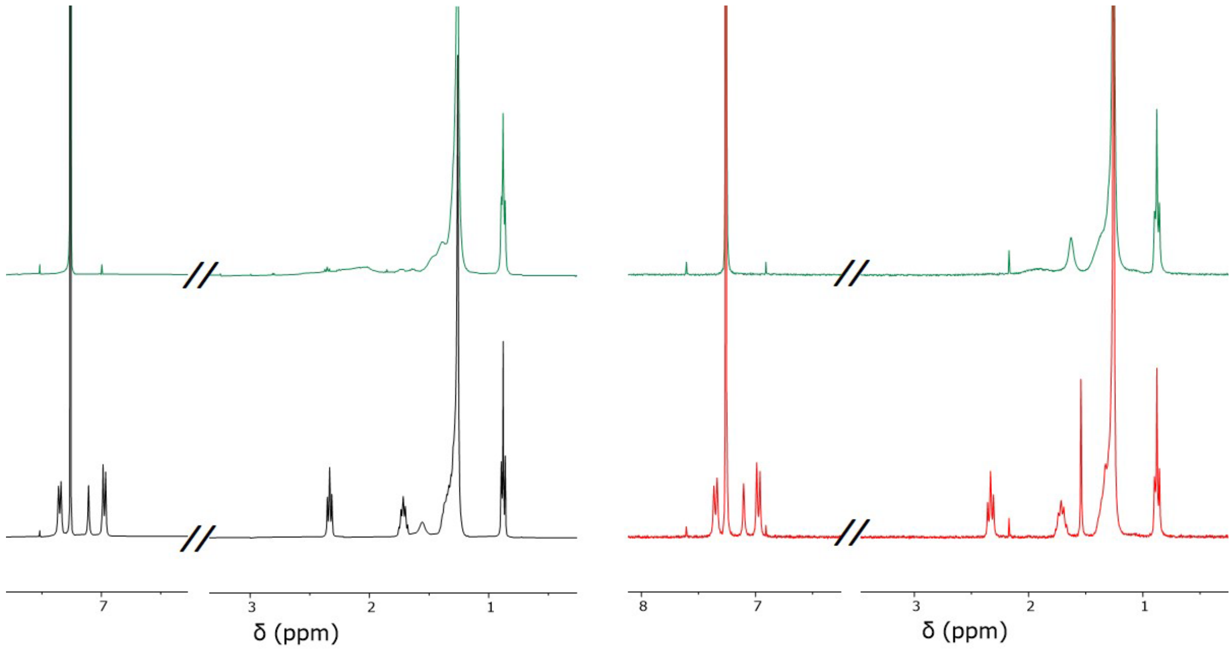

Figure S5 Left: ^{1}H NMR spectra of **TATA-C12** at 2.3 mM in $CDCl_3$ before (bottom) and after light irradiation (top) for 12 min. Right: ^{1}H NMR spectra of **TATA-C13** at 2.3 mM in $CDCl_3$ before (bottom) and after light irradiation (top) for 1 min.

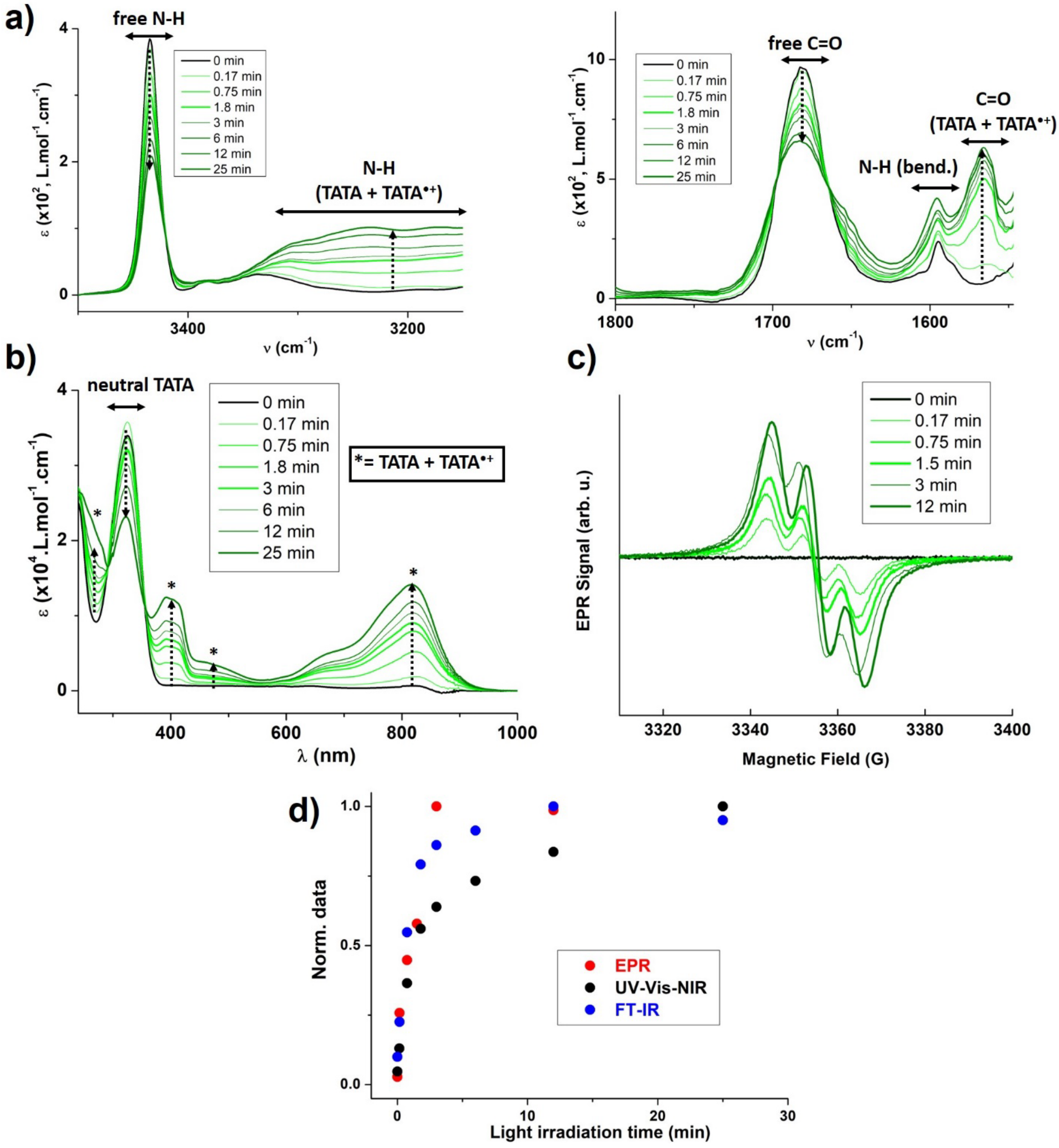

Figure S6 Assemblies in presence of light (intermediate regime, 2.3 mM TATA-C13 solutions in $CHCl_3$, 293 K). a) FT–IR absorption spectra before and after light irradiation (0-25 min), Zoom on the N─H region (left) and C=O regions (right). Arrows serve as a guide to the eyes for the evolution of the main bands upon increasing the irradiation time. b) UV-Vis-NIR absorption spectra before and after light irradiation (0-25 min). Arrows serve as a guide to the eyes for the evolution of the main bands upon increasing the irradiation time. c) EPR spectra before and after light irradiation (0-12 min), d) Intensities of EPR data, FT–IR data (band at 1566 cm^{-1}), and UV–Vis–NIR data (signal at 818 nm) normalized by their maximal intensity.

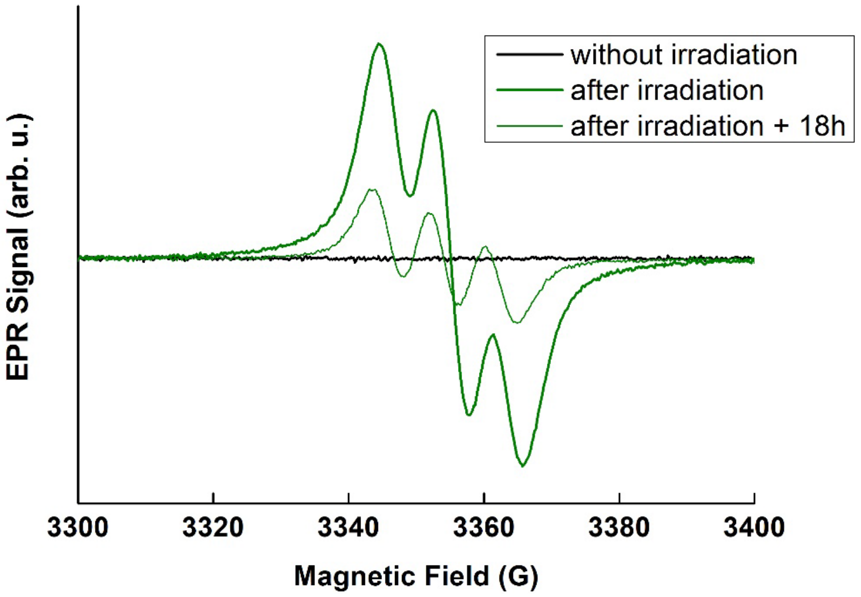

Figure S7 EPR spectra of **TATA-C12** at 2.3 mM in $CHCl_3$ before and after 12 min of light irradiation. The sample is kept 18h in the dark before being analysed again. The mol% of radicals decreased from 5.7% immediately after light irradiation to 1.5% after being aged in the dark for 18h (that corresponds approximately to the end of the SANS analysis). The preparation of this sample is identical to the one analysed by SANS and thus allows to estimate the number of radicals in the SANS sample to at least 1.5%.

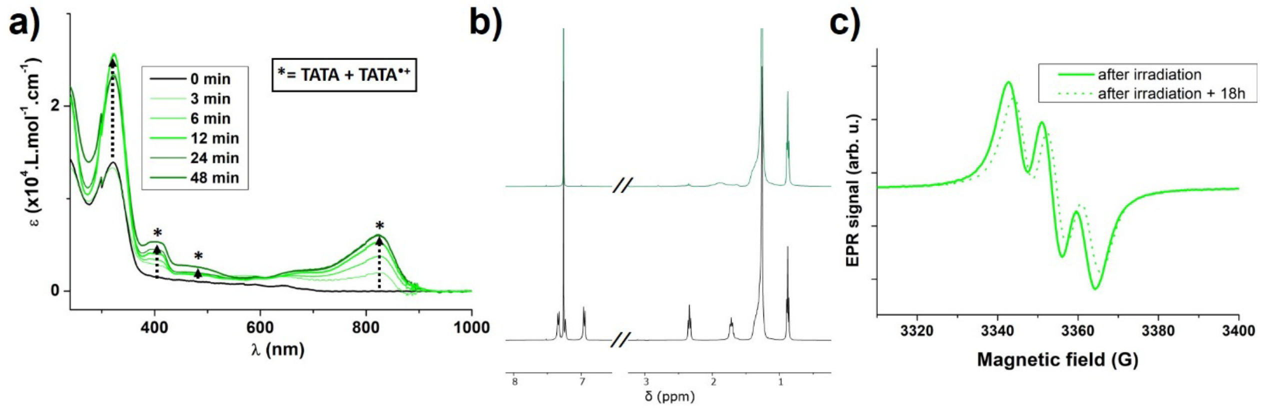

Figure S8 Assemblies in presence of light (concentrated solutions, 11.4 mM TATA-C12 solutions in $CHCl_3$, 293 K). a) UV-Vis-NIR absorption spectra before and after light irradiation (0-48 min). Arrows serve as a guide to the eyes for the evolution of the main bands upon increasing the irradiation time. b) ^1H NMR spectra in $CDCl_3$ before (bottom) and after light irradiation (top) for 12 min. c) EPR spectrum after 20 min of light irradiation. The sample was kept for 18h in the dark before being analysed again. The mol% of radicals decreased from *ca.* 1.5% immediately after light irradiation to 1.3% after being aged in the dark for 18h (that corresponds approximately to the end of the SANS analysis). The preparation of this sample is close to the one analysed by SANS and thus allows to estimate the number of radicals in the SANS sample to at least 1.3%.

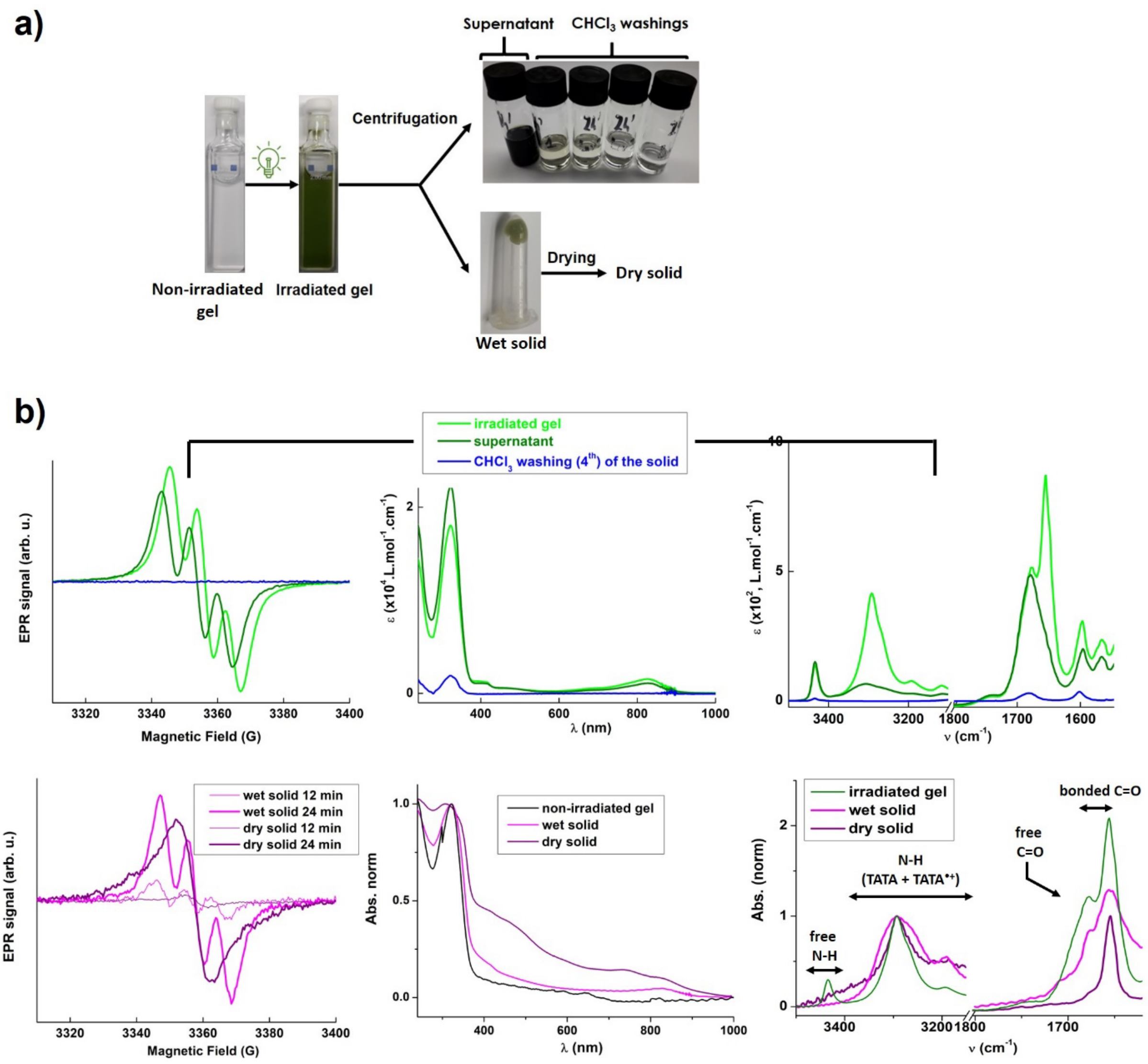

Figure S9 a) Pictures of the different steps leading to the isolation of wet and dry solids from a 11.4 mM solution of **TATA-C12** in $CHCl_3$. b) Top: EPR (left), UV-Vis-NIR absorption (middle) and FT-IR absorption (right) analyses of the starting irradiated gel (24 min of irradiation) and of the intermediate phases (supernatant and $CHCl_3$ washing). Bottom: EPR (left), UV-Vis-NIR absorption (middle) and FT-IR absorption (right) analyses of the wet and dry solids. Solids isolated from 11.4 mM solution of **TATA-C12** in $CHCl_3$ irradiated for 12 min were only analyzed by EPR. Even if the number of radicals cannot be quantified in these solids, the low signal-to-noise ratio observed for the 12 min-solids suggests a far lower number of radicals in these solids relatively to the 24-min solids. UV-Vis-NIR absorption (middle) and FT-IR absorption (right) analyses are normalized to the absorbance at 322 nm and 3292 cm^{-1}, respectively.

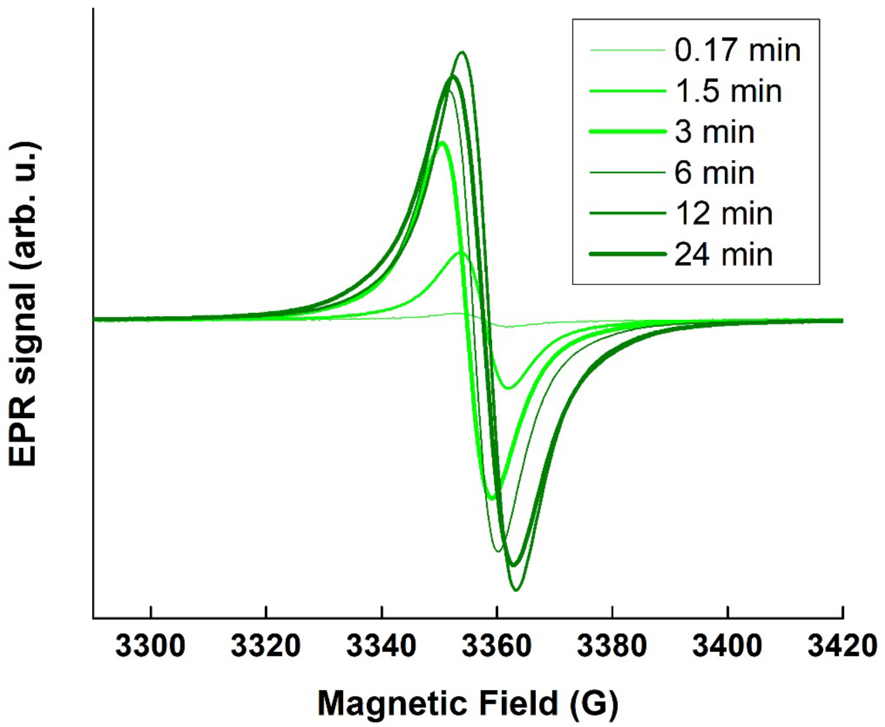

Figure S10 EPR spectra of films obtained by drop coasting of irradiated solutions of **TATA-C13** at 2.3 mM in $CHCl_3$ (0-24 min).

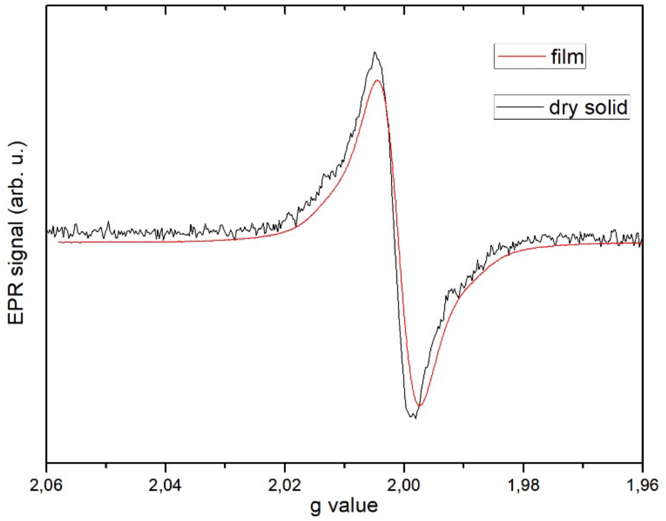

Figure S11 Compared EPR spectra of films and dry solids obtained from solutions of **TATA-C12** in $CHCl_3$.

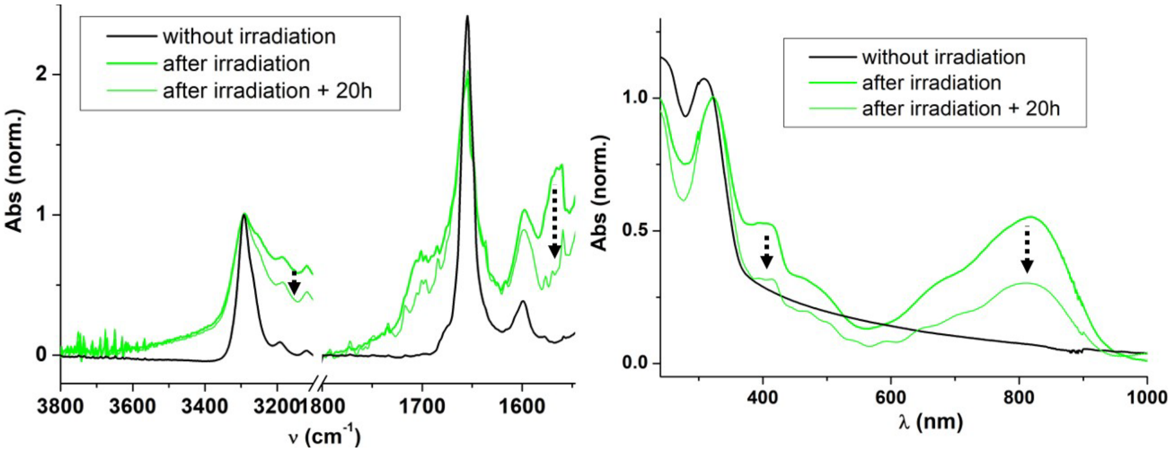

Figure S12 FT-IR (left) and UV-Vis-NIR (right) absorption spectra of films obtained by drop coasting of irradiated solutions of **TATA-C12** at 2.3 mM in $CHCl_3$ for 12 min. The sample is kept for 20h in the dark before being analysed again. The spectra are compared with that of the film obtained from a non-irradiated solution. Arrows are guide to the eye to indicate the decrease of the bands associated with TATA•+ species.

NMR spectra

TATA-C12

^{1}H NMR (THF-d_8, 300 K)

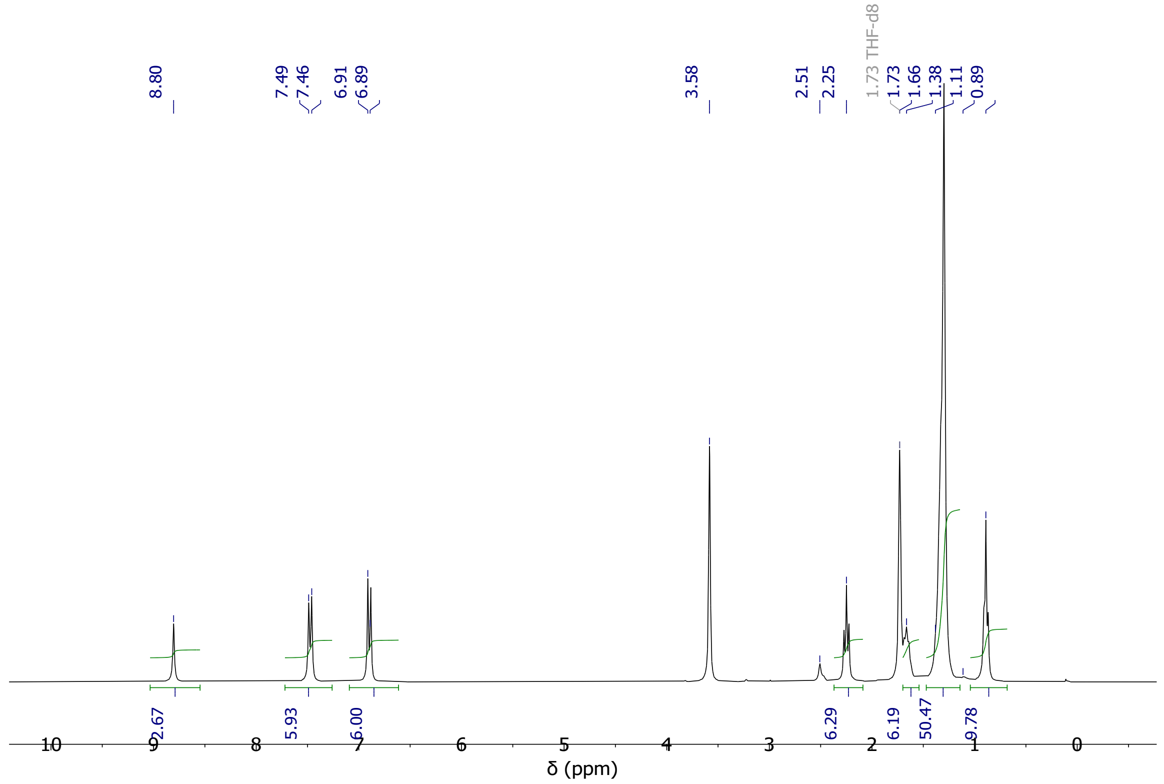

$^{13}C\{^{1}H\}$ NMR (THF-d_8, 300 K) DEPT135

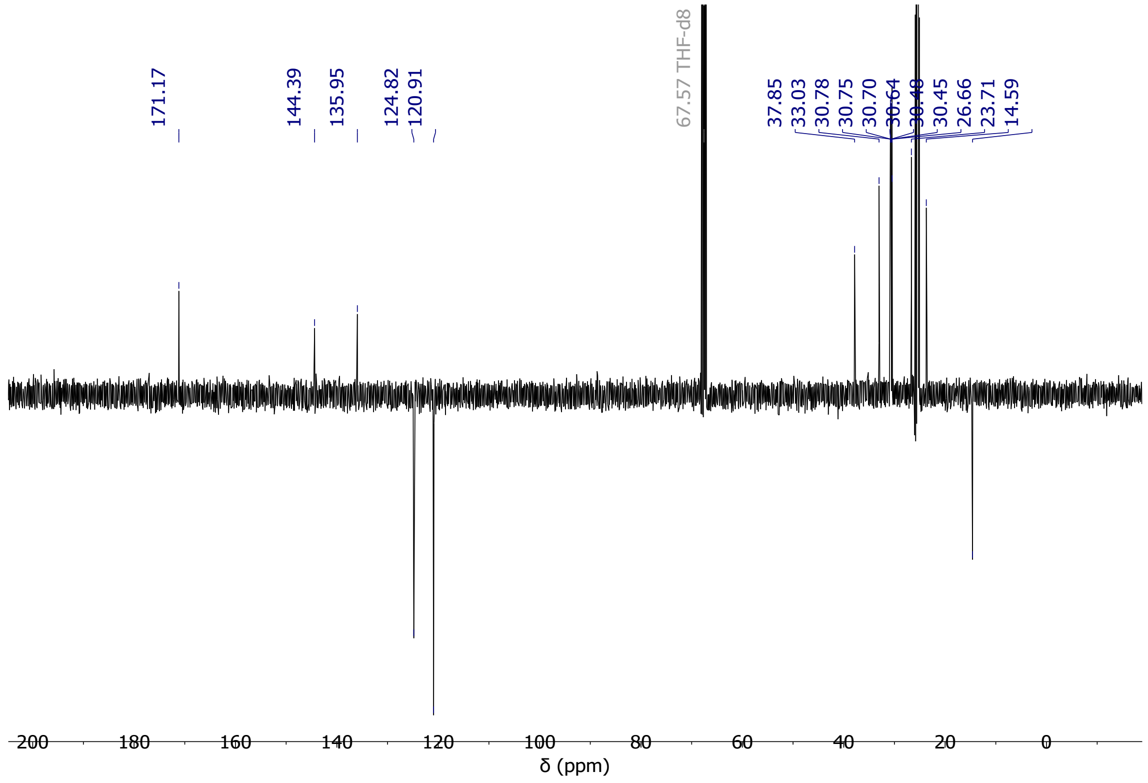

TATA-C13

^{1}H NMR (THF-d_8, 300 K)

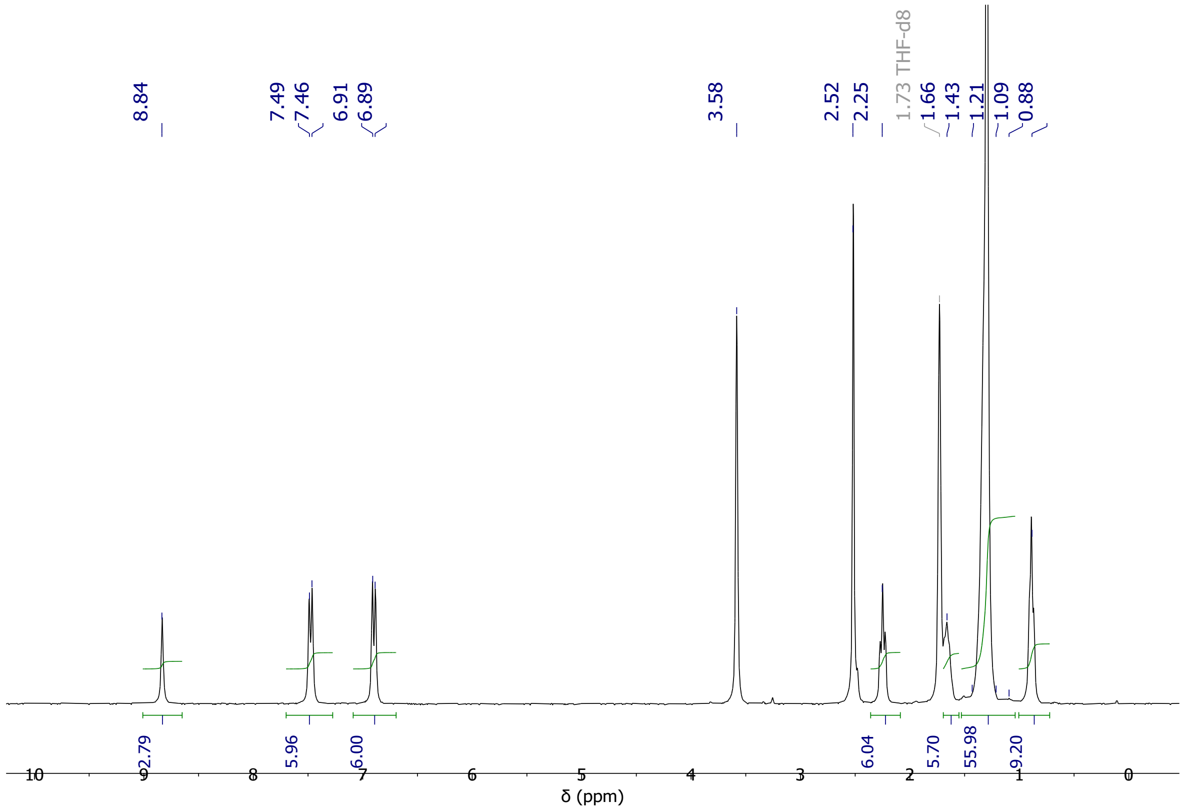

^{13}C{^{1}H} NMR (THF-d_8, 300 K) DEPT135

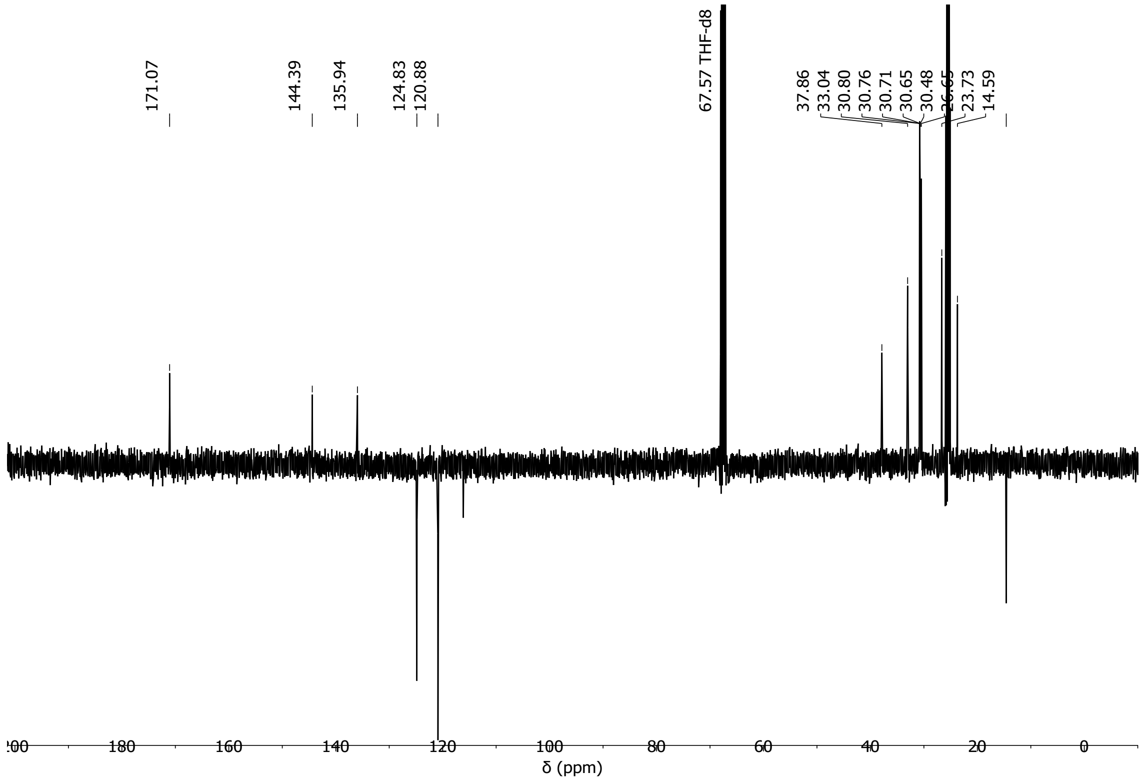

Supplement: Supplementary file 1 — Supporting Information [file CHEM-29-0-s001.pdf]
